# Trafficking of siRNA precursors by the dsRBD protein Blanks in *Drosophila*

**DOI:** 10.1093/nar/gkaa072

**Published:** 2020-02-06

**Authors:** Volker Nitschko, Stefan Kunzelmann, Thomas Fröhlich, Georg J Arnold, Klaus Förstemann

**Affiliations:** 1 Genzentrum & Department Biochemie, Ludwig-Maximilians-Universität, 81377 München, Germany; 2 Laboratory of Functional Genome Analysis, Ludwig-Maximilians-Universität, 81377 München, Germany

## Abstract

RNA interference targets aberrant transcripts with cognate small interfering RNAs, which derive from double-stranded RNA precursors. Several functional screens have identified *Drosophila blanks/lump (CG10630)* as a facilitator of RNAi, yet its molecular function has remained unknown. The protein carries two dsRNA binding domains (dsRBD) and *blanks* mutant males have a spermatogenesis defect. We demonstrate that *blanks* selectively boosts RNAi triggered by dsRNA of nuclear origin. Blanks binds dsRNA via its second dsRBD *in vitro*, shuttles between nucleus and cytoplasm and the abundance of siRNAs arising at many sites of convergent transcription is reduced in *blanks* mutants. Since features of nascent RNAs - such as introns and transcription beyond the polyA site – contribute to the small RNA pool, we propose that Blanks binds dsRNA formed by cognate nascent RNAs in the nucleus and fosters its export to the cytoplasm for dicing. We refer to the resulting small RNAs as blanks exported siRNAs (bepsiRNAs). While bepsiRNAs were fully dependent on RNA binding to the second dsRBD of blanks in transgenic flies, male fertility was not. This is consistent with a previous report that linked fertility to the first dsRBD of Blanks. The role of *blanks* in spermatogenesis appears thus unrelated to its role in dsRNA export.

## INTRODUCTION

The formation of double-stranded RNA (dsRNA) requires the presence of two RNA strands with complementary sequence. In *Drosophila*, these can arise via RNA-directed RNA polymerase activity during replication of certain RNA viruses in the cytoplasm (1,2) or by convergent transcription of DNA or through independent sense and antisense transcription from repetitive DNA loci (3). Furthermore, genes encoding long, self-complementary RNA molecules can provide endogenous substrates for Dcr-2. Invertebrates and plants make ample use of cytoplasmic double-stranded RNA for antiviral defense (4–9) and gene regulation (10–15). The Dicer enzymes process dsRNA into small interfering RNAs (siRNAs) that serve as specificity subunits in the RNA-induced silencing complex (RISC) and induce post-transcriptional degradation of cellular transcripts or viral genomes (16,17). Many of the cellular transcripts that are targeted for degradation by endogenous siRNAs derive from transposable element insertions. In addition, small RNAs including piRNAs (18–21) may enter the nucleus, identify cognate nascent transcripts and induce epigenetic silencing of the corresponding loci. The small RNA silencing system thus makes an important contribution to genome surveillance and stability.

Based on two decades of genetic, bioinformatic and biochemical work we have a detailed understanding of the canonical RNA interference (RNAi) pathway starting from dsRNA (10,22–23). First, the Dicer enzymes cleave long dsRNA into 21-mer siRNA precursors. These Dicer products are then loaded into RNAi effector proteins from the Argonaute family, where the siRNAs serve as specificity subunits and direct post-transcriptional silencing. This process is controlled at multiple steps (24) including the interpretation of the relative base-pairing stability at either end (25). For genome surveillance, we are beginning to understand the processes that govern the generation of dsRNA through convergent transcription of endogenous siRNA-generating loci. In contrast to pre-miRNAs, which are exported via Exportin-5 (26) or XPO1 (27), it is currently unclear whether dsRNA that forms in the nucleus is also processed there—followed by export of the siRNAs—or whether the dsRNA is exported to the cytoplasm and enters the canonical RNAi pathway. In a genome-wide screen, we had identified the dsRNA binding domain protein (dsRBP) Blanks as a factor that is required for efficient silencing triggered by dsRNA synthesis at DNA breaks in *Drosophila* cells (28). Previous studies had also identified *blanks/CG10630* as an RNA silencing factor (29–31) and indicated a role in chromatin biology (32,33), but did not propose a mechanism.

Here, we demonstrate that Blanks only stimulates silencing triggered by dsRNA that is generated in the nucleus. Blanks does not associate with Dcr-2 and it is not required for Ago2-loading of small RNAs. However, Blanks binds dsRNA, shuttles between nucleus and cytoplasm and likely facilitates the export of dsRNA for cytoplasmic dicing. We present a series of strongly *blanks*-dependent endo-siRNA loci at sites of convergent transcription, which we refer to as blanks-exported siRNAs or bepsiRNAs.

## MATERIALS AND METHODS

### Cell culture and dsRNA treatment

**Figure 1.**
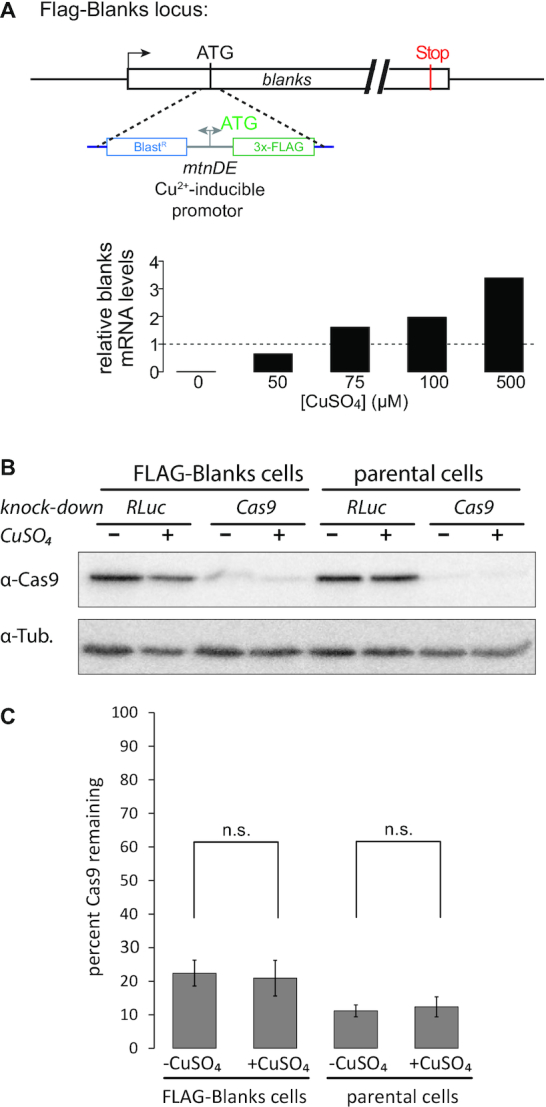
Blanks is not required for cytoplasmic RNAi. (**A**) Cartoon drawing of the modified Flag-blanks locus (top); transcription of the gene is now controlled by the copper-inducible *mtn* promoter contained in our marker/tag cassette. If all alleles of the targeted gene are modified, then gene activity can be fully controlled by the addition or omission of copper in the culture medium (bottom, normalized to *blanks* levels in non-edited cells). (**B**) We used the inducible Flag-blanks cell line to test the efficiency of cytoplasmic dsRNA-triggered RNA interference as a function of the presence or absence of *blanks*. We chose the *cas9* gene as a target because it is constitutively expressed in these cells as well as their progenitors and there should be no secondary effects resulting from the loss of this heterologous gene. A non-specific dsRNA targeting *Renilla* luciferase was used as a control (RLuc). (**C**) Quantification of the Western Blots from B; the Cas9 protein signal was normalized to the tubulin loading control, then the ratio between specific and control-knockdown was calculated. The graph shows the average of three independent biological replicates ± Standard Deviation (SD), n.s.: not significantly different (Student's *t*-test).

Cell culture experiments and generation of the genome-edited cell lines were performed as previously described (34,35). For the shut-down experiments, we first selected for integration of the Blasticidin^R^-mtnDE_promoter_-Flag_3_ cassette. Then, the cell population was diluted and cultured in the presence of 50% conditioned medium to derive single-cell clones. Finally, we used PCR with primers spanning the insertion site to verify the absence of non-modified loci. Prolonged culturing without CuSO_4_ in the culture medium results in nearly complete inactivity of the edited locus (Figure 1A and (35)). We induced Flag-Blanks expression by adding 200 μM CuSO_4_ to the cells for 96 h unless mentioned otherwise. The C-terminal Flag-tag on Blanks, R2D2 and Act5C was introduced as previously described (34), primer sequences are available in supplementary Table S1.

For the knock-down of cas9 in Figure 1, the cells were seeded at a density of 0.5 × 10^6^ cells/ml and 1 μg/ml dsRNA was added directly to the culture medium. The anti-cas9 antibody was obtained from Cell Signaling Technology (clone 7A9-3A3).

### Blanks-Dcr2 IP

Cells were harvested and washed twice in PBS before lysing them in 150 mM KAc pH 7.4, 30 mM HEPES pH 7.4, 5 mM MgAc, 1 mM DTT, 15% glycerol, 1% tergitol and 1 tablet of protease inhibitor per 10 ml (complete mini EDTA-free, Roche). Lysis was further facilitated using a Bioruptor (Diagenode, 20 cyles: 20 s ON, 20 s OFF). The lysate was cleared by centrifugation before it was incubated with Protein G Dynabeads (Invitrogen) that had been coated with 1 μl of anti-FLAG M2 (Sigma) per 10 μl beads. The beads were washed twice with 150 mM KAc pH 7.4, 30 mmM HEPES pH 7.4, 5 mM MgAc, 0.1% tergitol and twice with 150 mM KAc pH 7.4, 30 mmM HEPES pH 7.4, 5 mM MgAc. The beads were boiled in SDS loading dye for analysis on a SDS-PAGE and subsequent Western Blot performed as previously described (36). Antibodies used for the Western Blot were α-FLAG-HRP M2 (Sigma), α-Strep-tag II-HRP (IBA) or anti-Dcr2 (a kind gift of M. Siomi).

Details on our immunoprecipitations for mass spectrometry are given in the supplementary information.

### β-Elimination/deep sequencing

The β-elimination and deep sequencing library preparation were performed as previously described (37) with the exception of using the ZR small RNA PAGE Recovery Kit (Zymo Research) for small RNA purification after the PAGE-steps. Sequencing was performed on an Illumina HiSeq instrument at LAFUGA (Gene Center, LMU Munich, Germany). Sequencing reads were demultiplexed and 3′ adapter trimmed with custom scripts (available on request). The reads for the analysis of the miRNA-, transposon and endo-siRNA-mapping sequences were size selected for 20–23 nt long reads and mapped with bowtie.

To create the list of loci with the potential for convergent transcription, we first extracted all gene coordinates from the *Drosophila* genome annotation file (version 6.02 .gff, downloaded from Flybase) with linux command line tools (grep -w ‘FlyBase’ dmel-all-no-analysis-r6.02.gff | grep -w ‘gene’ | grep -v ‘parent_type’ |cut -f1,4,5,7,9 >gene_coordinates_r6_02.bed), then created a list of overlapping genes with opposite orientation extended by 300 nt on the 3′-end using bedtools window (38) (bedtools window -l 0 -r 300 -sw -Sm -a gene_coordinates_r6_02.bed -b gene_coordinates_r6_02.bed > overlapping_3p300_extended_genes_r6_02.bed). This list contained two entries for every potential overlap (one from the sense and one from the antisense-running gene), we thus generated a non-redundant set by restricting the orientation of the first gene to sense only. Finally, we simplified the name field to only the FBgn number with a custom Perl script.

The sequencing libraries were first size-selected to 21-mers and then filtered by mapping to the *Drosophila* transposon consensus sequences (no mismatch allowed), retaining only the non-matching reads. This dataset was then mapped to the *Drosophila* genome (version 6.02) with no mismatch allowed, only reporting reads that map uniquely. The overlap of this analysis with the regions of convergent transcription (see above) was determined by applying bedtools intersect with the –c option. We normalized differences in sequencing depth by calculating the ppm values relative to all genome matching reads in the filtered dataset.

The analysis of the bepsiRNAs in the testes samples was performed by first selecting only 21 nt long reads and removing the transposon-matching reads as described above. The remaining reads were mapped to all extended gene regions (2 kb extended precomputed set based on release 6.02, downloaded from Flybase and reduced to 150 nt on each side) using bowtie. The total number of *Drosophila* genome matching reads was used to normalize for differences in sequencing depth between the libraries in all cases. The sequencing data has been deposited at the European Nucleotide Archive (ENA) under accession number PRJEB32123.

### Importazol assay

The Blanks-GFP or H2Av-GFP cells were diluted to 2 × 10^6^ cells/ml and treated either with 200 μM Importazol (Sigma) or an equal volume of DMSO. The localization of the GFP fusion proteins was quantified 16 h later by visual inspection with a fluorescence microscope; images were acquired on a Zeiss LSM710 confocal microscope. A live cell DNA-counterstain was performed by adding 1 μl Hoechst 33342 (10 μg/ml) to 10 μl resuspended cells.

### RNA binding assay

The RNA preparation of the 23 nt long double-stranded siRNA forming 21 bp and a 2 nt 3′ overhang on each end and the RNA-binding assay was performed as previously described (39).

### Molecular biology

The plasmid backbone for the expression of the blanks variant transgenes in flies was pKF63 (40) that was modified by adding an *attB* site by oligonucleotide annealing and cloning the product into the *Nde*I site. The blasticidin resistance from plasmid pMH3 (34) was excised and cloned into the *Nde*I site in front of the attB-site. The resulting plasmid was digested with *Bam*HI and *Not*I and the GFP-insert was replaced with the blanks CDS generated by PCR with *Drosophila* cDNA as a template. The cDNA sequence contains polymorphisms and corresponds to the sequence described with GenBank ID: AY119201.1. In comparison to the reference sequence, this sequence variant harbors a 8 aa deletion of D^69^-R^76^, a 4 aa deletion of V^90^-D^93^ followed by an aa exchange (D^94^ → N), a 2 aa insertion of KE after K^104^, three single aa exchanges (R^150^ → H, L^190^ → M, G^194^ → E) and two silent point mutations (A^435^ → G, A^816^ → G). In the remainder of this manuscript, the numbers of nucleotides and amino acids refer to the corresponding position in the reference sequence. The FLAG-NLS sequence and the mutations in the dsRBD2 were introduced via the PCR primers into the inserts of the corresponding plasmids.

The inserts for the recombinant expression vectors were created by PCR from the vectors created above with the ‘*5*′ *wildtype*’ and either the ‘*3*′ *wt-dsRBD2*’ or ‘*3*′ *mut-dsRBD2*’ primer. The primers used for the PCR were ‘*5*′ *wildtype*’ and ‘*3*′ *wt-dsRBD2*’. The PCR products were cloned into the pGEX-6P-1 backbone using *Bam*HI and *Not*I.

All primer sequences are available in supplementary Table S1 (note the separate tabs in the Excel file).

### Generation of transgenic and rescue fly lines

For generation of the transgenic lines the plasmids with a mini-white marker were injected into embryos and inserted into an attp2 site via the ϕC31 integrase method (41). The injected flies were crossed with a *w[1118]* stock and the resulting red-eyed flies (from the transgene) were used for crosses to generate the homozygous transgene flies.

For the generation of the rescue line, mutant virgins (y^1^ w*; +; Mi[MIC]blanks^MI10901^) were crossed with males from the homozygous blanks transgene lines. F1 virgins were crossed with males from the balancer stock *yw; +; D/TM3,Sb*. Through meiotic recombination both the blanks mutant and the blanks transgene can end up on the same chromosome. Flies with red eyes were backcrossed with the balancer stock to isolate the chromosome carrying the transgene with the mini-white marker. Brothers and sisters from the backcross were used to generate a homozygous stock and the presence of the *blanks^MI10901^* mutant was confirmed by PCR.

### Fertility assay

One male of the fly line whose fertility was assessed was crossed with two *yw* virgins at 25°C (aged for two days after hatching) and those were then allowed to lay eggs for 4 days. Pupae were counted 9–10 days after that.

### Testes dissection and RNA isolation

About 150 testes were hand-dissected for each RNA isolation and library preparation. The dissected testes were crushed in 500 μl TRIzol reagent (Ambion) using a pestle. RNA was then isolated according to the manufacturer's protocol and directly used for library preparation.

### Recombinant protein expression and purification

The expression plasmids were transformed in BL21 (DE3) pLysS cells. The expression culture was inoculated at OD_600_ = 0.1 in 1 l LB with ampicillin and chloramphenicol that was supplemented with 0.5% glucose. The culture was grown at 25°C until OD_600_ = 0.6 and then induced with 1 mM IPTG for 2 h. Cells were harvested, washed once in PBS and frozen in 8 ml lysis buffer (50 mM Tris pH 7.0, 150 mM NaCl, 5 mM DTT, 10 μg/ml lysozyme, 0.1 U/ml DNase I, 1% Triton and 1 tablet of protease inhibitor on 10 ml (complete mini, Roche). After thawing lysis was facilitated using a Bioruptor (Diagenode, 30 cyles: 30 s ON, 30 s OFF). The lysate was cleared by centrifugation and by passing it through a syringe filter before loading on a GSTrap HP column (GE Healthcare). The column was washed with 10 ml lysis buffer, 5 ml high salt buffer (50 mM Tris pH 7.0, 1,5 M KAc pH 7.0, 5 mM DTT) and again with 10 ml lysis buffer. Elution was done in 5 ml 50 mM Tris pH 8.0, 1,5 M, 5 mM DTT and 20 mM reduced glutathione. The elution fractions containing the protein were pooled and incubated with 2 U of PreScission protease (GE Healthcare) over night at 4°C to cleave off the GST-tag. The cleaved protein was diluted in 3 volumes of 50 mM HEPES pH 7.0 and loaded on a HiTrap SP HP ion exchange column (GE Healthcare). The column was washed with 10 ml 50 mM HEPES, pH 7.0 and eluted in 50 mM HEPES pH 7.0 with a gradient from 0 to 1000 mM NaCl. Fractions containing the protein were pooled and diluted in 3 volumes of 100 mM KAc pH 7.4, 10 mM HEPES pH 7.4, 2 mM MgAc, 5 mM DTT and then concentrated using Amicon Ultra Centrifugal Filters with a 10 kDa cutoff.

## RESULTS

### Blanks is not required for cytoplasmic RNAi

Our genome-wide RNAi screen for the generation of DNA-break derived siRNAs identified *blanks* as a stimulating factor (28). We now present our work on the mechanism of action of this double-stranded RNA binding protein (dsRBP). First, we tested whether cytoplasmic RNA interference, triggered by soaking S2 cells in medium containing dsRNA, requires *blanks*. To avoid a sequential combination of RNAi, which complicates interpretation, we created a clonal cell line with CuSO_4_-inducible Flag-Blanks expression via genome-editing (*mtnDE* promoter inserted, schematic shown in Figure 1A, top). Due to the insertion of a selection marker and the *mtnDE* promoter in front of the blanks coding sequence (CDS), omission of copper sulfate from the culture medium leads to transcriptional shutdown of the *blanks* locus modified at all alleles (Figure 1A, bottom). We chose a heterologous gene as the target of RNAi in order to avoid any potential feed-back regulation; conveniently, all our genome-edited cell lines continue to express the *S. pyogenes* Cas9 protein. We thus added dsRNA targeting the transgenic *cas9* mRNA to the cell culture medium (without transfection reagent, the standard approach for RNAi in cultured *Drosophila* cells) of cells that either expressed *blanks* or not. Four days later, we determined the remaining Cas9 protein levels as a function of Blanks expression by western blotting (Figure 1B). Whether or not *blanks* was expressed, the RNAi pathway could be efficiently programmed by dsRNA delivered to the cytoplasm. We conclude that while we identified blanks as an important player for an RNAi response triggered by dsRNA generated in the nucleus, the protein is fully dispensable for a purely cytoplasmic RNAi response.

### Blanks does not interact with Dcr-2

**Figure 2.**
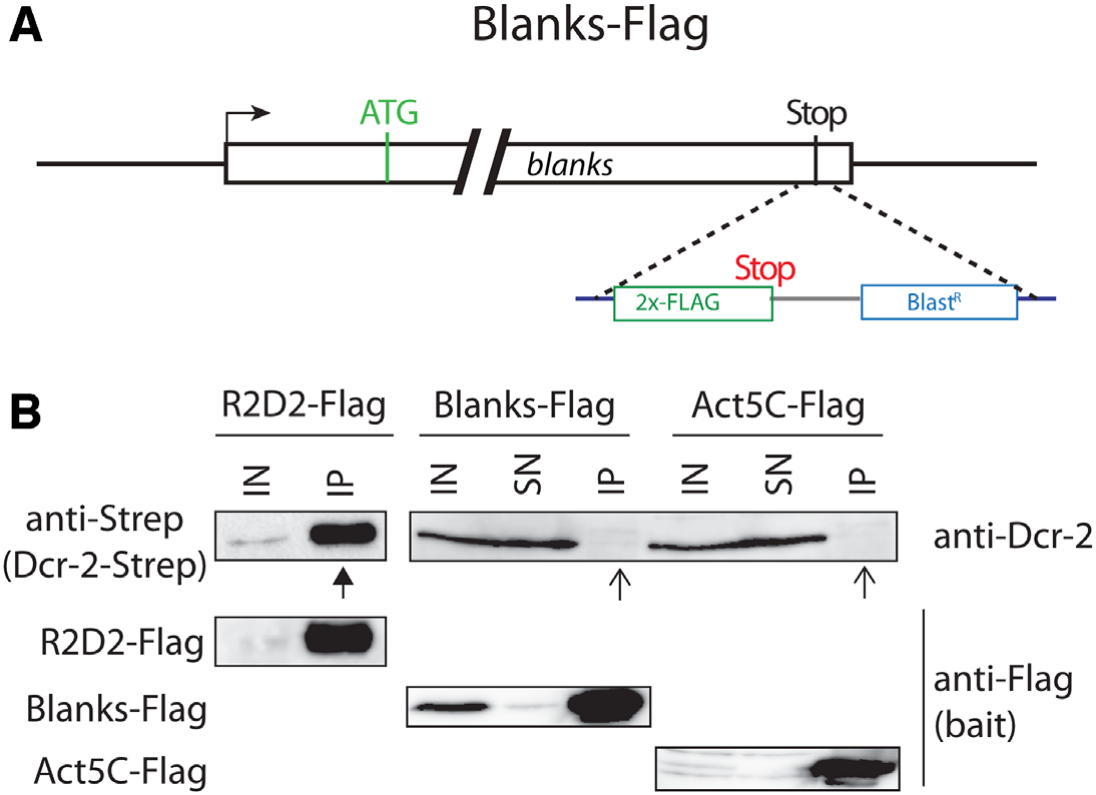
Blanks and Dcr-2 do not form a stable complex. (**A**) Cartoon drawing of the Blanks-Flag locus; in this case, the tag is inserted at the C-terminus, hence transcription occurs at endogenous levels driven by the gene's natural promoter. (**B**) Co-immunoprecipitation experiments from whole cell lysates do not indicate a stable complex between Blanks and Dcr-2. We also generated knock-in cell lines expressing Flag-tagged versions of R2D2 and Actin5C as positive and negative controls, then prepared whole cell lysates and immunoprecipitated the Flag-tagged proteins. Association of Dcr-2 was detected via a monoclonal anti-Dcr-2 antibody (Blanks-Flag and Actin5C-Flag samples, mAB 8–59, a kind gift of M. Siomi) or via an anti-Strep tag antibody (the R2D2 cells also harbor a Dcr-2-Strep-tag knock-in). While the co-immunopreciptiation of Dcr-2 was readily detectable with R2D2 (filled arrowheads), no association was seen with Blanks or Actin5C (open arrowheads).

The tandem dsRBD domain architecture of Blanks resembles the Dcr-2 partner proteins Loqs as well as R2D2. To probe for a potential association between Blanks and Dcr-2, we performed genome-editing to generate a C-terminally tagged Blanks-Flag fusion protein. This way we can retain regulation by the endogenous promoter and avoid overexpression artifacts (Figure 2A). We then immunoprecipitated the Flag-tagged protein as well as analogously modified R2D2-Flag as a positive control from knock-in cell extracts. An Actin5C-Flag cell line served as background control. We could not detect any co-purification of Dcr-2 with either Blanks or Actin5C (Figure 2B, open arrowheads). In contrast, Dcr-2 substantially co-purified when extracts from R2D2-Flag cells were employed (Figure 2B, filled arrowhead). Our results are consistent with prior studies, which indicate that Dcr-2 resides in the cytoplasm (42) while Blanks is localized predominantly in the nucleus and Dcr-2 could not be detected among the associated proteins (30,31). In conclusion, the currently available experimental data does not support a complex between Blanks and Dcr-2. A direct contribution of *blanks* to the dicing of long dsRNA into siRNAs is thus unlikely.

### A subset of endogenous natural antisense transcript (NAT) derived siRNAs depends on blanks

The identification in several screens suggested that *blanks* augments the siRNA response triggered by endogenous sources of dsRNA. We constructed small RNA sequencing libraries from the genome-edited shut-down cell lines we generated for Blanks (Figure 1A) and Dcr-2 (35) to assess which endogenous, dsRNA generating loci show a dependence on *blanks*. To focus on genic sites of siRNA origin, we removed all siRNAs that showed a perfect match to the collection of *Drosophila* transposon consensus sequences (Flybase) and then restricted the alignments for uniquely matching 21-mer reads.

For further analysis, we compiled a list of sites with the potential to be convergently transcribed. The annotated mRNA 3′-ends generally correspond to the mapped poly-adenylation site(s). Yet, the transcribing RNA polymerase continues a certain stretch downstream until it is either stripped off the DNA by Xrn2 (according to the so-called torpedo-model) (43) or terminates due to a conformational change (44). Because these highly unstable transcripts could contribute to the pool of nuclear dsRNA, we extended all genes by 300 nt at their 3′-end and listed all genes that overlap in opposite orientation, but not necessarily involving the 3′-extension. The compiled list comprises 4366 distinct candidate regions, of which 1748 produced >5 ppm of sequence reads when normalized to sequencing depth (corresponding to at least 11 siRNA reads in the wt library).

**Figure 3.**
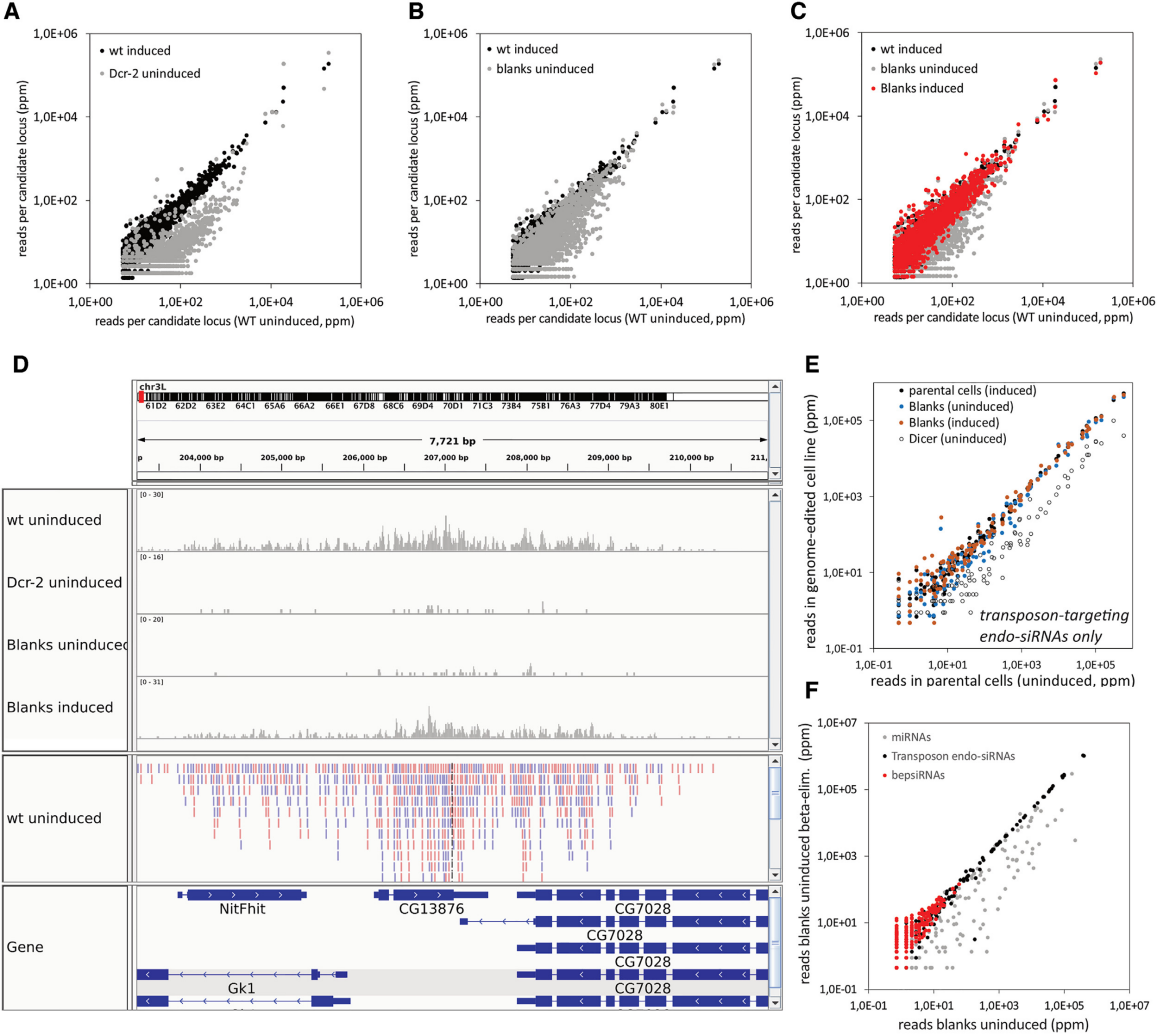
*blanks* dependent siRNAs are generated at sites of convergent transcription. Small RNAs were isolated from S2-cells with copper-inducible *blanks* and *Dcr-2* alleles (see Figure 1 for a detailed example), sequenced, filtered for transposon-matching reads and the respective read counts are depicted as parts (reads) per million (normalized to the total number of genome matching reads in each library). The term ‘induced’ refers to culturing in the presence of 200 μM CuSO_4_ while ‘uninduced’ refers to culture in the absence of CuSO_4_ (also referred to as ‘shut-down’ in the manuscript). (**A**) The majority of convergent gene loci give rise to a moderate amount of *dcr-2* dependent siRNAs (4366 loci analyzed). (**B**) A subset of these loci also requires *blanks* (see also Supplementary Table S2). (**C**) Re-expression of blanks by adding copper to the culture medium of our Flag-Blanks cells (‘induced’) recovered the expression of the small RNAs. In essence, the blanks-dependent siRNA loci are the grey points that remain visible below the diagonal that is populated by the wt (black) and induced blanks (red) samples. (**D**) The salient features of a blanks-dependent siRNA generating locus are illustrated by this genome browser image. The coverage tracks in the upper part were adjusted to differences in sequencing depth and thus faithfully convey different siRNA levels, the corresponding libraries are indicated on the left. We also present the annotation of individual siRNAs for the wild-type sample below the coverage with color-coded orientation (pink: 5′→3′ left to right, blue: 5′→3′ right to left). The gene annotation on the bottom shows two pairs of convergent genes. (**E**) The amount of transposon-targeting endogenous RNAs depends on *dcr-2* but not on *blanks*. (**F**)To determine whether the remaining *blanks*-dependent siRNAs (bepsiRNAs) are mis-loaded into Ago1 we sequenced oxidized small RNA libraries. While miRNAs do not carry the protective 2′-O-methyl modification at the 3′-end that is characteristic for Ago2-loading, the transposon-targeting and the remaining bepsiRNAs are protected and hence correctly loaded.

The vast majority of these small RNA generating regions showed a clear dependence on Dcr-2 (Figure 3A), indicating that many sites of natural antisense transcription (*cis*-NAT loci) do produce *bona fide* siRNAs, albeit at a low level. When the small RNAs from our *blanks* shut-down cell line were analyzed, we saw that a subset of these regions generated fewer siRNAs in the absence of *blanks* (Figure 3B). Re-expression of *blanks* rescued the abundance of the corresponding siRNAs, confirming the contribution of *blanks* to their biogenesis (Figure 3C). Based on the ratio of small RNA reads in the library from wt cells to the reads in the *blanks* shutdown library, we compiled a list of 528 *blanks* dependent siRNA generating genomic loci (Supplementary Table S2, arbitrary cutoff: ratio ≥ 5).

Figure 3D shows the salient features of a blanks-dependent siRNA locus as a genome browser (IGV) view. First, according to the initial filter in our analysis, the region has convergently transcribed genes that overlap or are in close proximity. Second, the siRNAs from the locus are strongly diminished upon transcriptional shutdown of *dcr-2* as well as *blanks* but their abundance is rescued by re-induction of *blanks* expression (Figure 3D, coverage traces). Third, the siRNAs that map to the locus are found in both sense and antisense orientation—consistent with their dependence on *dcr-2* and the potential for convergent transcription (Figure 3D, feature trace shown for wt cells). Fourth, the siRNA generating region extends significantly beyond the annotated transcript end/poly-adenylation site (Figure 3D, gene annotation trace for convergent genes *CG13876* and *CG7028* on the right side). Finally, the siRNA generating part of the convergent overlap is neither limited to the 3′-region nor to exons; rather, the dsRNA seems to form at the level of the nascent transcript. For example, the transcript of the *NitFhit* gene is fully contained within the first intron of the *Gk1* locus but runs in the opposite orientation (Figure 3D, gene annotation trace – left side).

### How do the blanks-dependent siRNA generating loci differ from previously described siRNA-generating cis-NAT loci?

Okamura and colleagues have compiled a list of convergent, natural antisense transcripts that generate siRNAs (45). These siRNAs arise almost exclusively from genes whose 3′-regions converge. In particular, the siRNAs map only to the overlaps up to, but not beyond, the annotated polyA-site. Manual inspection of these loci demonstrated that the previously annotated siRNAs depend on *Dcr-2* but not *blanks*. However, at these sites as well, there are *blanks*-dependent siRNAs at a much lower abundance that extend well beyond the annotated 3′-overlap on either side (e.g. *mus205* or *Sin3A* locus, Supplementary Figure S1). Apparently, two distinct mechanisms can lead to siRNA formation at convergent genes. One is dependent on both *Dcr-2* and *blanks* and acts on nascent transcripts. In contrast, the second one apparently acts on processed transcripts and is solely dependent on *Dcr-2*.

### Are transposon-targeting endo-siRNAs dependent on blanks?

In the somatic cells of *Drosophila*, an abundant class of endogenous siRNAs is targeting the transcripts of transposable elements (TE). They arise via bi-directional transcription through a process that is still mechanistically unclear. We thus wondered whether *blanks* was required for the biogenesis of TE-targeting siRNAs in cultured S2-cells. To sum up the reads in our libraries for each TE sequence, we mapped the full libraries to the TE consensus sequence set. Between libraries, the read counts were normalized to the number of uniquely matching, non-TE reads from our previous analysis. A scatter plot of the shut-down cell lines vs. the parental cells revealed, as expected, that the absence of Dcr-2 severely reduced the abundance of TE-targeting siRNAs (Figure 3E, open circles). In contrast, shut-down of *blanks* did not have a major effect on TE-targeting siRNAs (Figure 3E, compare e.g. the blue and orange dots).

### Blanks-dependent siRNAs are loaded into Ago2

Small RNA abundance can be measured in a relatively straightforward way by deep sequencing. However, this does not necessarily imply that the sequenced small RNAs are incorporated into the Argonaute effector complexes and thus fully functional. The loading of siRNAs into *Drosophila* Ago2 requires a RISC loading complex consisting of Dcr-2 with the dsRBD-containing proteins R2D2 (46) or Loqs-PD (39). We can assess the loading state of siRNAs by their susceptibility to a chemical modification: Once loaded into Ago2, the small RNA is 2′-*O*-methyl modified at the 3′-terminal nucleotide and thus lacks the vicinal diol needed for oxidation with periodate. In contrast, unloaded or Ago1-loaded small RNAs are not modified and thus depleted from small RNA libraries prepared after periodate treatment. Since the dsRBD arrangement of Blanks is reminiscent of R2D2 and Loqs, we tested whether Blanks participates in Ago2-loading of endogenous siRNAs and sequenced oxidized small RNAs from *blanks* shut-down cells. We found that Ago1-loaded miRNAs were efficiently depleted from the library, while TE-targeting siRNAs remained resistant, hence 2′-*O*-methyl modified and thus Ago2-loaded. Although the *blanks*-dependent siRNAs were obviously not abundant in *blanks* shut-down cells, the remaining RNAs showed no sign of mis-loading into Ago1 (Figure 3F). We conclude that *blanks* is not required for the loading of siRNAs into Ago2.

### Blanks shuttles between nucleus and cytoplasm

**Figure 4.**
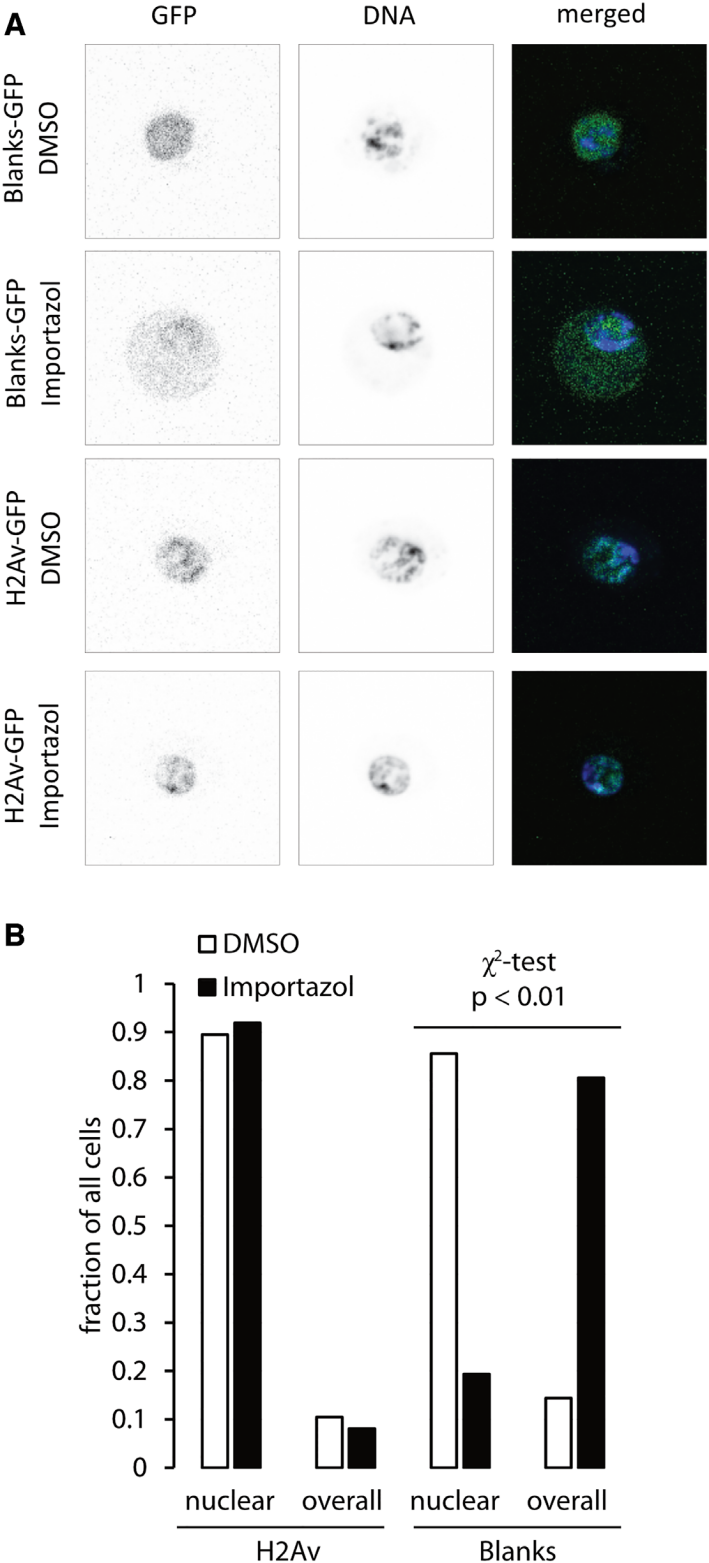
Blanks shuttles between nucleus and cytoplasm. (**A**) Fluorescence microscopy images of cells with Blanks-GFP and H2Av-GFP knock-ins. An example of the control (DMSO) and the Importazol-treated cells is shown for each. The DNA was stained with Hoechst33342. (**B**) Quantification of protein localization after treatment with the nuclear import inhibitor Importazol for 16 h. Cells were classified into either showing a predominantly nuclear or a whole-cell staining (‘overall’). The slides were prepared, anonymized and then analyzed by visual inspection using a fluorescence microscope. While the distribution of H2Av-GFP did not change during the course of Importazol treatment, the localization of Blanks changed from predominantly nuclear to mostly a whole-cell-staining.

We have demonstrated so far that Blanks is not required for purely cytoplasmic RNAi, consistent with its steady-state nuclear localization. Furthermore, Blanks does not form a complex with Dcr-2 and acts on dsRNA that is generated from convergent nascent transcripts in the nucleus. A common theme among all functional screens that identified *blanks/CG10630* as required for small RNA mediated repression is that DNA-based constructs were used to generate the silencing trigger in the nucleus. Could Blanks be an export-factor that facilitates export of dsRNA so that it can be processed by cytoplasmic Dcr-2? If so, then Blanks should not be constitutively nuclear but rather shuttle between nucleus and cytoplasm. Indeed, treatment of cells harboring a Blanks-GFP knock-in allele (Figure 4A), where localization of Blanks can be directly observed, with the nuclear import inhibitor Importazol resulted in an increased cytoplasmic fraction of Blanks. Control cells with an H2Av-GFP fusion did not show a changed distribution. This confirms that the perturbation of Blanks is due to blocked re-import rather than accumulation of newly translated protein in the cytosol (Figure 4B). A mass spectrometry based interactome analysis with extracts from the Flag-Blanks knock-in cell line revealed association with ribosomal and spliceosomal proteins, presumably mediated by binding of Blanks to the abundant structured RNA present in these complexes. In addition, we observed a significant enrichment of the nucleo-cytoplasmic transport factors Ran, Rcc1 (the Ran guanine nucleotide exchange factor) and Kap-α3 (an importin-α homolog) as well as the nucleoporin Mtor in the Flag-Blanks immunoprecipitations (see Supplementary Figure S2 and Supplementary Table S3 for full results). This is consistent with the nucleo-cytoplasmic shuttling of Blanks-GFP we observed via the Importazol treatment.

### The second dsRBD of blanks can bind dsRNA

The dsRBD is a small folded domain that can also evolve to mediate protein-protein, rather than protein-RNA, interactions. Blanks harbors two of these domains, but the first instance has diverged strongly from the consensus and is unlikely to bind dsRNA. We confirmed that recombinantly expressed Blanks protein does indeed bind to dsRNA *in vitro* and introduced inactivating point mutations into the second dsRBD as a control. In fact, the inactivating changes we introduced in the second dsRBD (K^301^K^302^→ AA) appear to be already present in the wild-type sequence of the first dsRBD instance in Blanks (V^165^N^166^ instead of KK at the corresponding position). We quantified binding to a 23 nt synthetic RNA ligand (21 bp and a 2 nt 3′-overhang on each end) by fluorescence anisotropy as previously described (39) (Supplementary Figure S3). Wild-type Blanks bound dsRNA with an affinity in the higher nanomolar range (*K*_D_ = 177 ± 22 nM, *n* = 3), consistent with the affinity of an individual rather than tandem dsRBDs for siRNA (39). The mutant protein bound dsRNA almost 4-fold less tightly (*K*_D_ = 666 ± 197 nM, *n* = 3). This effect strength of an introduced point mutation is comparable to observations with other dsRBP proteins (47). Blanks is thus not merely a dsRBD containing protein but does indeed use at least the second dsRBD instance to bind dsRNA.

### RNA binding proficiency of Blanks does not affect the male fertility defect of blanks mutants

**Figure 5.**
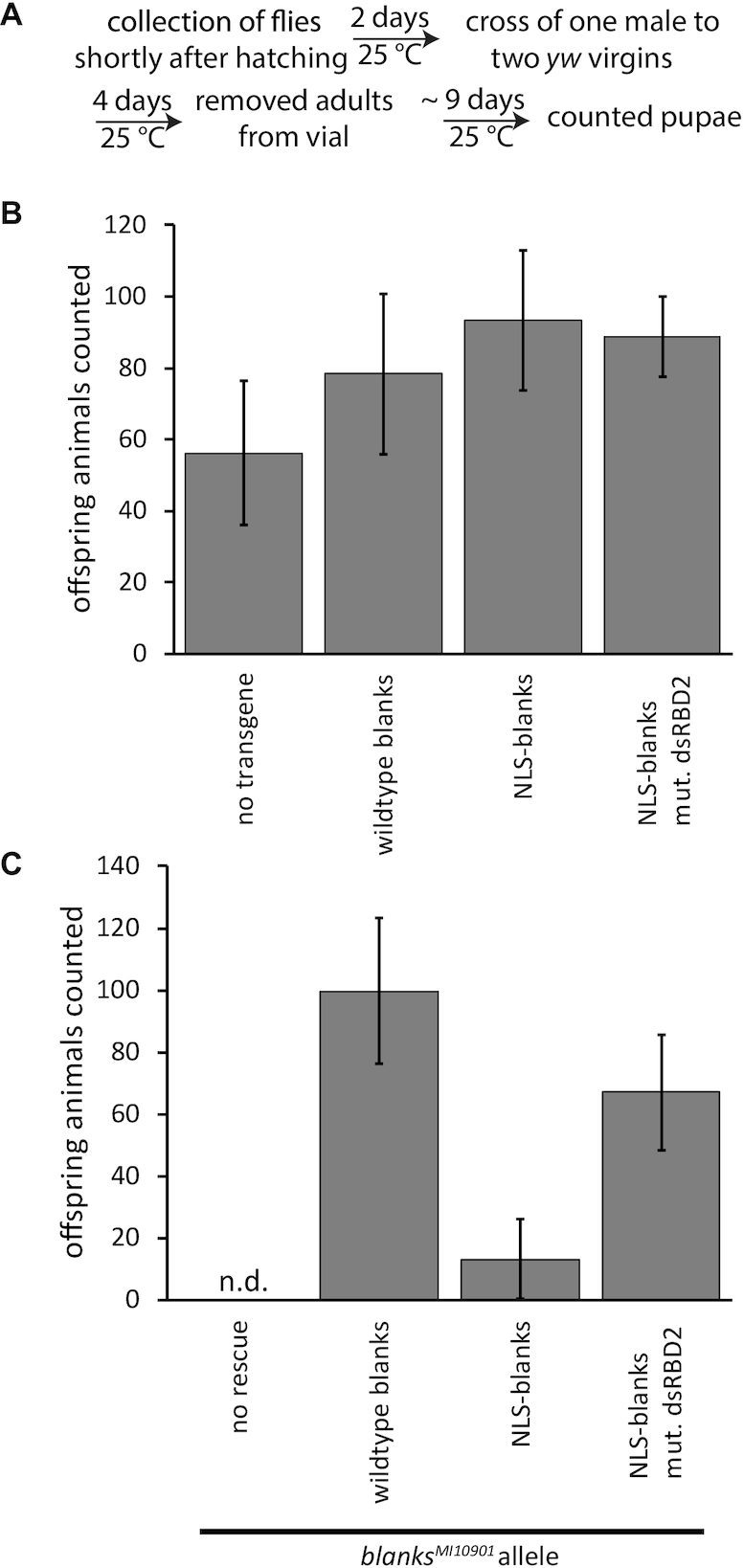
Fertility of male flies is influenced by localization and RNA-binding ability of Blanks. The bars indicate the average number of pupae, error bars represent the standard deviation (*n* = 5). (**A**) Flowchart of the fertility assay. (**B**) Ubiquitous expression of our *blanks*-transgenes has no dominant-negative effect on male fertility in a wild-type background. (**C**) The *blanks*-transgenes can rescue fertility; however, an increased nuclear retention via the appended NLS is detrimental if the blanks protein is competent for RNA binding. For the no rescue condition, zero pupae developed in all replicates (none detected, n.d.).

The *blanks* gene is not essential for viability of fruit flies but *blanks* mutant males are sterile, consistent with its predominant expression in testes (30,31). This infertility phenotype had previously been linked to the integrity of the first dsRBD of Blanks, while mutation of the second dsRBD had no effect on spermatogenesis (31). However, Sanders and colleagues introduced a mutation that potentially disrupts folding of the dsRBD rather than dsRNA binding specifically. We thus generated transgenic versions of *blanks* carrying the same point mutation in the second dsRBD that we had analyzed *in vitro*. We also increased nuclear retention by adding an additional nuclear localization sequence. First, we confirmed that our constructs do not show a dominant-negative effect on male fertility in the context of a wild-type *blanks* locus (Figure 5A, B and Table 1). Furthermore, both the wild-type transgene and the extra-NLS transgene with an inactivated dsRBD2 could efficiently restore male fertility when recombined onto a *blanks* mutant chromosome. Clearly, RNA binding by dsRBD2 of Blanks is not required for male fertility. In contrast, the Blanks protein with an extra NLS and a wild-type dsRBD2 only reached a partial rescue of fertility (Figure 5C and Table 1). We propose that this effect may to be due to excessive trapping of precursors for miRNAs or hairpin-RNAs in the nucleus (see below and Discussion).

**Table 1.** Summary of phenotypes with mutant *blanks* transgenes

	Male fertility	miRNA biogenesis	bepsiRNA biogenesis
blanks^MI10901^	**−**	**+**	**−**
blanks^MI10901^, wt Blanks	**+**	**+**	**+**
blanks^MI10901^, NLS-Blanks	**−**	**(+)**	**(+)**
blanks^MI10901^, NLS-Blanks + mutant dsRBD2	**(+)**	**+**	**−**
Blanks mutant dsRBD1 (Sanders *et al.* 2011)	**−**	**+**	(nd)
Blanks mutant dsRBD2 (Sanders *et al.* 2011)	**+**	(nd)	(nd)

### Increased nuclear retention of Blanks results in export defects of structured RNA

**Figure 6.**
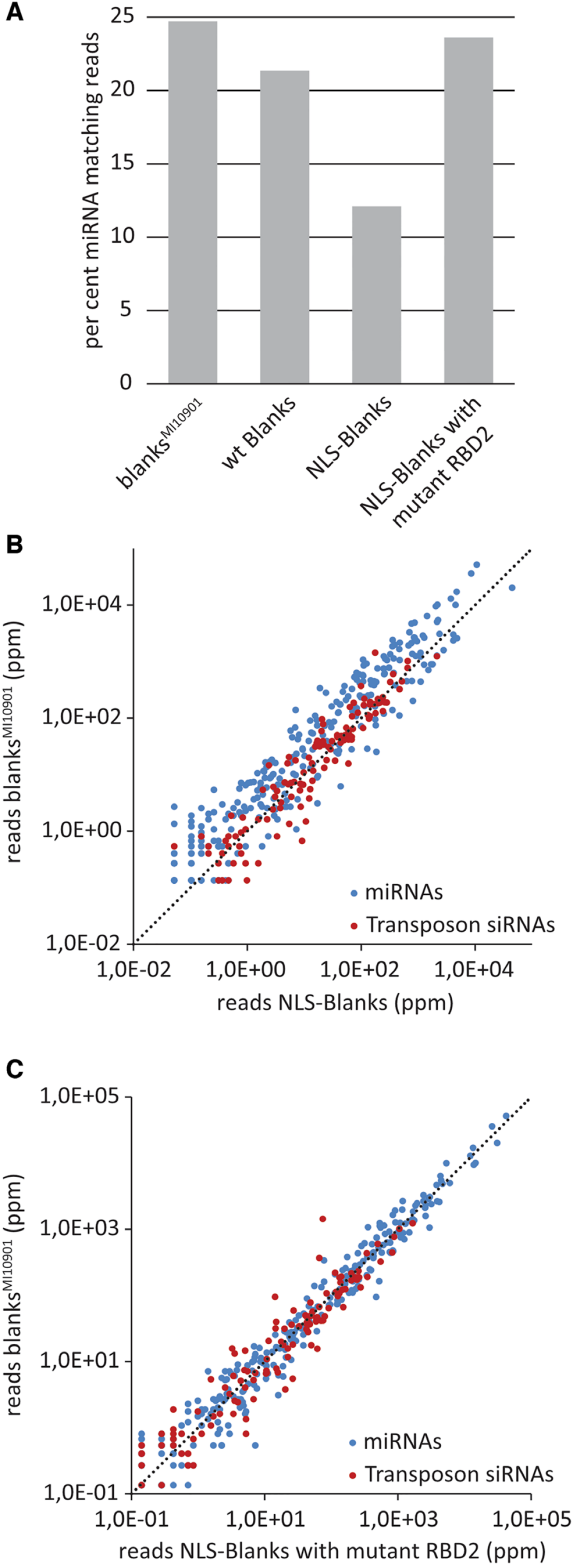
The effect of wild-type and mutant *blanks* transgenes on the small RNA profile in *blanks^MI10901^* mutant testes. (**A**) The relative proportion of miRNA-matching reads in among the small RNAs is not sensitive to the presence or absence of Blanks. However, expression of an RNA-binding proficient Blanks protein with an additional nuclear localization signal (NLS) reduced the abundance of miRNAs. (**B**) This reduction of miRNA matching reads is due to a generalized but moderate effect on all miRNAs (blue dots), rather than selectively affecting, e.g. only the highly expressed species. (**C**) If the Blanks transgene harbors an inactivating point mutation in the second dsRBD in addition to the NLS, the inhibition of miRNA biogenesis is not visible.

We concluded above that Blanks can function with nuclear dsRNA, likely upstream of Dcr-2 processing. One can envision that Blanks regulates chromatin and transcription or assists in the formation of dsRNA from single-stranded RNAs; alternatively, Blanks may facilitate the export of dsRNA for further processing in the cytoplasm. Increased nuclear retention of Blanks should increase a putative role in transcriptional regulation and/or improve any RNA-chaperone like function; in contrast, any export-related activity should be perturbed. We thus examined the small RNA repertoire of dissected testes (the tissue with highest blanks expression) from *blanks^MI10901^* mutant males and our rescue lines. We combined the *blanks^MI10901^* mutation with transgenic constructs for wildtype Blanks (referred to as wt in the remainder of the text) as well as NLS-Blanks with either a functional or a mutant dsRBD2. Consistent with the S2-cell data (Supplementary Figure S4), our analysis revealed that miRNAs were mostly unaffected by the absence of *blanks*. However, introduction of a *blanks* version with the additional NLS reduced the abundance of miRNA reads by half (Figure 6A). The reduction appears to depend on the ability of Blanks to bind the partially double-stranded miRNA precursor: Under the same conditions, the corresponding dsRBD2-mutant protein did not show an inhibitory effect on the miRNA pathway. In contrast, transposon-matching siRNAs remained mostly unaffected (Figure 6B, C and Table 1). This observation is consistent with a model where the overexpressed, NLS-Blanks protein spends more time in the nucleus where it binds excessively to the double-stranded part of miRNA-precursors.

### Blanks-dependent siRNAs depend on convergent transcription in vivo

**Figure 7.**
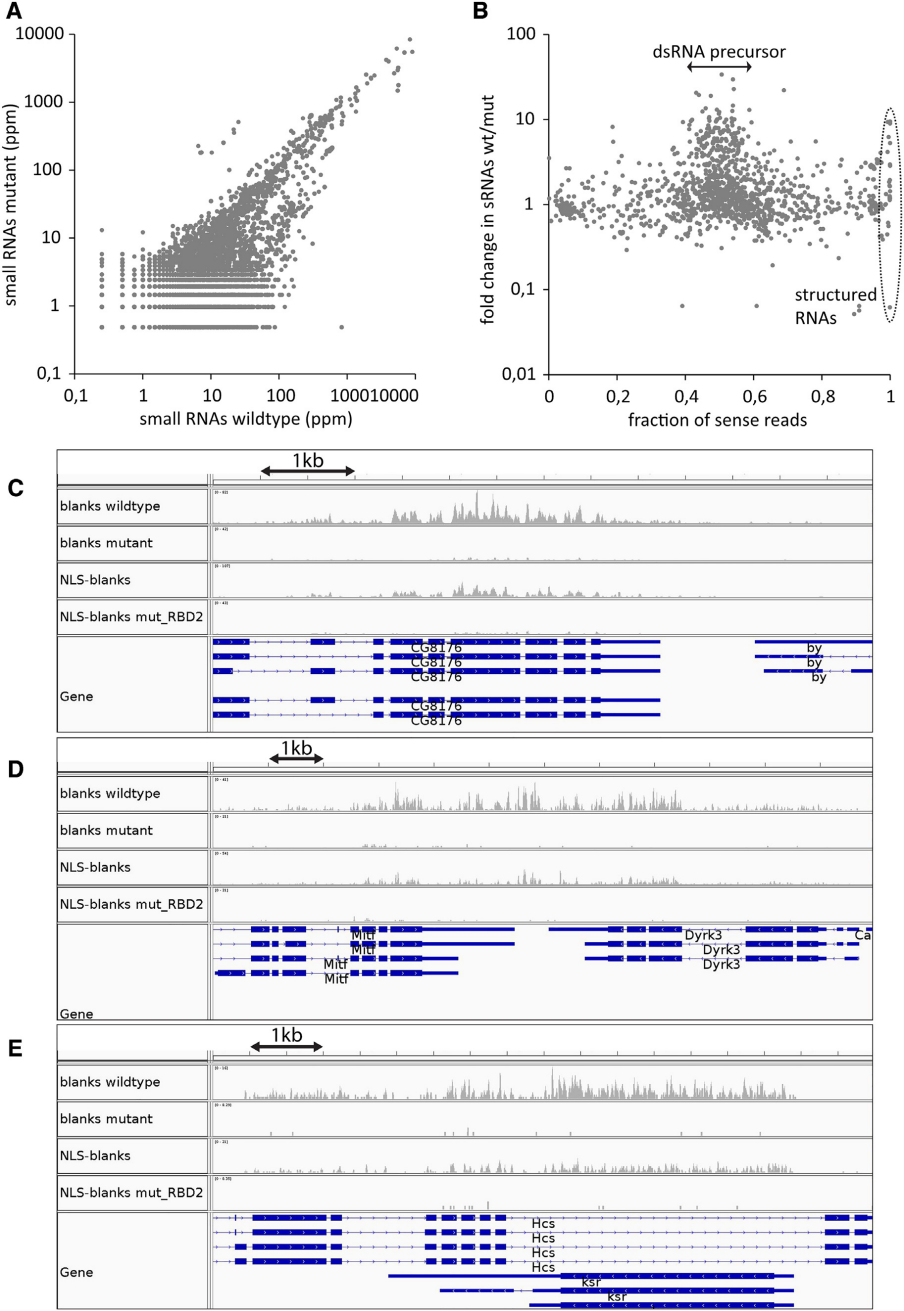
Analysis of Blanks exported siRNAs (bepsiRNAs) in fly testes. (**A**) Unbiased analysis of 21-mer siRNA reads mapping to all annotated transcripts extended by 150 nt on each end. Depicted are the sequence reads (normalized to total genome matching) for *blanks^MI10901^* mutant animals and mutants rescued with a wild-type blanks protein (referred to as wt). A substantial number of loci generates *blanks*-dependent siRNAs (below the diagonal); the small group of genes with increased expression (above the diagonal) are members of the 825-*oak* gene family, a known source of hairpin RNAs. (**B**) We calculated the change between wt and *blanks^MI10901^* mutants for each annotated gene and then plotted this relative to the fraction of reads that map in sense orientation at the respective locus. Most blanks-dependent siRNA loci generate siRNAs with sense and antisense orientation in roughly equal amounts, indicating that they derive from a dsRNA precursor (central region, fold change >>1). Consistent with the S2-cell data in Figure 3, not all dsRNA-derived siRNAs are *blanks*-dependent (central region, fold change ∼1). In addition, several structured non-coding RNAs (tRNA and rRNA) give rise to blanks-dependent siRNAs, which match exclusively in sense-orientation. In this plot, the fraction of sense reads was calculated based on the read-counts of the wild-type library. The supplement contains an overlay with a calculation of the fraction of sense reads from the blanks^MI10901^ library. (**C–E**) Genome browser views for *CG8176* and *by* (C), *Mitf* and *Dyrk3* (D), *CG10508* and *CG12975* (E). The coverage traces were adjusted to account for the differences in sequencing depth.

To evaluate the importance of nucleo-cytoplasmic shuttling for blanks-dependent siRNAs, we analyzed the blanks-dependent siRNA coverage of annotated genes. To obtain a global view, we did not restrict this analysis to convergent gene pairs but rather included all annotated genes in the analysis. Furthermore, we extended the genes by 150 nt on either side, rather than 300 nt only on the 3′-end, and quantified the small RNA reads mapping to each gene. To focus on blanks-dependent siRNAs, we removed all TE-matching siRNA reads (thus also removing potential 21 nt long piRNA degradation products). In the wildtype library, siRNA-levels in genic regions were overall higher than in the *blanks* mutant (Figure 7A), though we cannot fully exclude a bias due to differences in sequencing depth (wt library: 1755972 uniquely mapping 21-mer reads, blanks^MI10901^: 756901 uniquely matching 21-mer reads). For a (semi)-quantitative description, we therefore only retained those loci that gave rise to at least 10 reads in each of our libraries. If Blanks acts on dsRNA, then the resulting siRNAs should show roughly equal amounts of sense- and antisense-matching reads. When we plotted the change between wild-type and *blanks* mutant for each locus as a function of the corresponding sense/antisense ratio, we saw that the genes with a roughly equal sense and antisense representation preferentially showed a dependence on *blanks* (Figure 7B, central region). This is consistent with our observations in S2-cells and the *in vitro* binding studies. The 10-read cutoff criterion leads to the exclusion of many genes that show very low siRNA levels in the *blanks* mutant testes – likely including some of the strongest examples of *blanks*-dependent siRNA loci. We therefore directly counted the number of loci giving rise to small RNAs with siRNA-like sense and antisense representation and found that there was a clear increase in the wild-type library relative to the *blanks* mutant. Thus, the generation of *blanks*-dependent siRNAs is also widespread *in vivo* (Supplementary Figure S5).

Upon manual inspection, many of these loci were found at sites of convergent transcription (e.g. Figure 7C–E). In some cases, the *blanks*-dependent siRNAs mapped preferentially to the exonic regions (e.g. *CG8176*, Figure 7C or *Dyrk3*, Figure 7D), while in other cases introns clearly contributed to the dsRNA precursor (e.g. *Hcs* intron, Figure 7E). In order to directly compare the *blanks-*dependent siRNA loci between flies and S2-cells, we analyzed the fly sequencing libraries exactly as the S2-cell libraries before (only unique mappers, focused analysis of loci with potential for convergent transcription). We then ranked the loci by the wt/mutant ratio and selected all loci that had a ratio ≥5 (Supplementary Table S4). Out of the 4366 regions analyzed, 528 fulfilled this criterion in the S2-cell libraries (12.1%) and 600 fulfilled it in the fly testes libraries (13.7%). There were 209 regions common to both (corresponding to 39.6% of the S2-cell regions and 34.8% of the fly testes regions), indicating that *blanks* functions in a similar manner in both cell types.

### Shuttling and dsRNA binding by Blanks are important for siRNA generation

Our analysis has revealed that blanks is required for the generation of small RNAs at convergently transcribed regions. We analyzed the sequencing data obtained from flies with an extra NLS on otherwise wild-type Blanks and found that 666 loci met the cutoff-criterion of a rescue/mutant ratio ≥5. Overall, there was a very good agreement between the two datasets. Nonetheless, it appears that increased nuclear retention lowers the siRNA yield from more active regions while it may slightly increase the yield from the regions with lower activity (Supplementary Figure S6A). The regions displayed in Figure 7C–E are examples of rather active loci and the traces reflect the reduced yield seen in the rescue with NLS-blanks. It thus appears that in particular the more abundant dsRNA precursors become trapped in the nucleus. The less abundant ones might benefit from the presumably higher nuclear concentration of NLS-Blanks, which may outweigh the negative effect of a prolonged nuclear retention. Importantly, siRNA generation was entirely dependent on the RNA binding capacity of dsRBD2. When the corresponding data was analyzed, we found that now only 73 loci met the ratio cutoff-criterion and overall the ratios no longer correlated between a rescue with wild-type blanks protein and the rescue with the blanks variant carrying inactivating point mutations in dsRBD2 (Supplementary Figure S6B).

In conclusion, we identified widespread generation of siRNAs at convergent genes at a low to moderate level. The production, but not the loading, of these siRNAs depends on *blanks*. Specifically, Blanks must be able to bind dsRNA with its second dsRBD and to freely shuttle between nucleus and cytoplasm for full activity. The most parsimonious interpretation is that Blanks facilitates export of siRNA precursors. Hence, we refer to the small RNAs that need the assistance of Blanks as ‘Blanks exported siRNAs’ or bepsiRNAs.

## DISCUSSION

**Figure 8.**
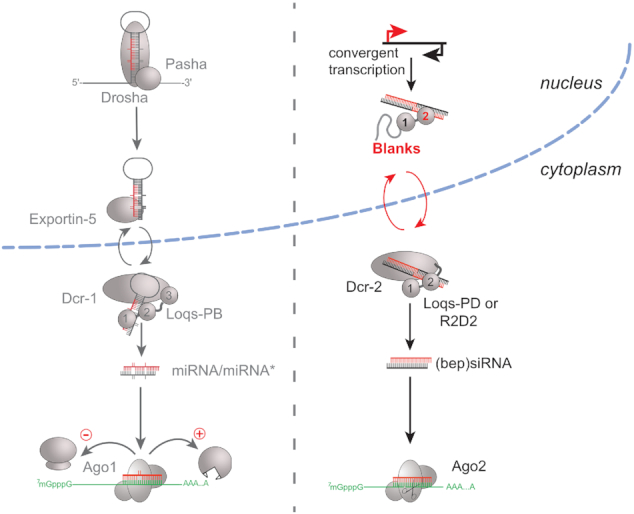
We propose that Blanks serves as a link between nuclear dsRNA and the export machinery. While the nuclear export of miRNA precursors has been characterized (left), export of dsRNA to the cytoplasm for processing by Dcr-2 is less well understood. We interpret our results that one export route for endo-siRNA precursors is through association with Blanks, a protein that binds to dsRNA with its second double-stranded RNA binding domain (dsRBD) and shuttles between nucleus and cytoplasm (right). The dsRBDs of Loqs, R2D2 and Blanks are indicated as spheres and numbered according to their position within the protein.

RNA interference (RNAi) is a phenomenon where aberrant RNA is detected, converted to double-stranded RNA (if necessary) and processed into siRNAs, which then target the aberrant RNA for degradation. In addition to cytoplasmic dsRNA, e.g. from a replicating virus, aberrant nuclear transcripts can also be funneled into a siRNA response. For example, siRNAs repress endogenous retroviruses (ERV) and transposable elements in somatic and germ line cells of *Drosophila* (48–52). While the nuclear export pathway for pre-miRNAs is well understood (53–55), the fate of nuclear dsRNA is unclear. One hypothesis is that a fraction of *Drosophila* Dcr-2 is present in the nucleus (56) but the immunostaining of Dcr-2 protein in the nucleus with a monoclonal antibody does not exceed background levels (42); nuclear Dcr-2 is thus scarce if not absent. On the other hand, the nuclear-localized form of Tombusvirus p19, a viral RNAi suppressor that tightly binds duplex 21 nt siRNAs, perturbed heterochromatin formation in transgenic flies; this argues that double-stranded Dcr-2 products can play a nuclear role in flies (57). The export of dsRNA from the nucleus into the cytoplasm should require an adaptor protein that links the dsRNA based on its structure, but not the particular sequence, to the Ran-GTP/GDP cycle. The double-stranded RNA binding domain (dsRBD) is a protein module that recognizes the structure of dsRNA specifically but not the nucleotide sequence (58). In this manuscript, we demonstrate that the *Drosophila* dsRBD protein Blanks binds to dsRNA, interacts with proteins from the nuclear import/export pathway, shuttles between nucleus and cytoplasm and that biogenesis of endo-siRNAs at regions of convergent transcription is perturbed in *blanks* mutant flies. A straightforward *interpretation* of these facts is that Blanks serves as an export adapter, which links the binding of dsRNA with the nuclear export machinery (summarized in Figure 8). A more complicated model would postulate that Dcr-2 enters the nucleus in cells that do not express Blanks and that the 21nt siRNA products of Dcr-2 require only Exportin-5 to reach the cytoplasm. Finally, our results do not formally exclude that Blanks binds dsRNA in the nucleus, escorts it towards the nuclear pores but does not transit to the cytoplasm together with its cargo. However, such a hand-over mechanism from Blanks to e.g. Exportin-5 would have to be perturbed for pre-miRNAs, the well-known substrates for Exportin-5, by the additional NLS we appended to Blanks (nuclear trapping of pre-miRNAs, Fig. 6a).

Drosophila *blanks/lump/CG10630* has previously been identified as a small RNA silencing factor (29–31) and is required for efficient siRNA generation at a DNA double-strand break (28). Despite the convincing scores that *blanks* achieved in the screens, no mechanistic information on Blanks’ molecular function in small RNA silencing is available. A first indication is that the requirement for Blanks is dependent on the site of dsRNA generation: According to our analysis, Blanks is not required for cytoplasmic processing of exogenous dsRNAs into siRNAs and the targeting of cognate messages during RNAi (Figure 1). Consistent with this observation, we were unable to demonstrate an interaction of Blanks with Dcr-2 (Figure 2, Supplementary Table S3). Gerbasi and colleagues also have not found any association with Dcr-2 and rather demonstrated that Blanks co-purifies with a set of proteins including the RNA helicases Rm62, Me31B and the ribonuclease Xrn2. Although we note that this complex has not yet been reconstituted with recombinant proteins, the described associations are not mutually exclusive with our trafficking model. Our results differ, however, with respect to the role of *blanks* in cytoplasmic RNAi triggered by dsRNA. It is conceivable that this was caused by different strategies for dsRNA administration: While in our experiments the dsRNA trigger was introduced by soaking and thus endocytosis, Gerbasi *et al.* transfected the dsRNA along with the reporter gene plasmids. Perhaps a significant fraction of this dsRNA reached the nucleus along with the DNA and thus became dependent on *blank*s for export to the cytoplasm (30).

Our small RNA sequencing from a genome-edited cell line with inducible *blanks* expression revealed a specific population of small RNAs whose biogenesis is facilitated by Blanks (Figure 3). The siRNA populations are comprised of roughly equal amounts of sense and antisense-matching species, consistent with their occurrence at sites of potential convergent transcription. While the genome annotation defines a gene's 3′-end at the site of polyadenylation, the underlying transcription units can apparently be extended by hundreds or even thousands of nucleotides in many cases (e.g. Supplementary Figure S1), thus giving rise to large windows for bepsiRNA generation. A direct interpretation of this observation is that Blanks binds dsRNA that forms between nascent transcripts in the nucleus. Because the small RNAs were also Dcr2-dependent and Ago2-loaded, they represent a subset of *bona fide* endo-siRNAs. Interestingly, a set of previously identified (45) natural antisense gene pairs (*cis*-NATs) generates siRNAs at much higher levels from the region of overlap that is upstream of the polyA-site in each gene, arguing that the dsRNA precursor is formed between processed mRNAs. These siRNAs depend on *dcr-2* but not on *blanks*. Perhaps in these cases, the mature mRNA molecules form dsRNA between their complementary regions only once they are in the cytoplasm and thus have no need for nuclear export facilitation of dsRNA. Loading of the bepsiRNAs as well as transposon-targeting siRNAs into Ago2 was not impaired when Blanks was absent. Accordingly, the role of Blanks must be upstream of dsRNA processing by Dcr-2.

We extend the observation of a predominantly nuclear localization of Blanks at steady-state (30) by demonstrating that Blanks shuttles between the nucleus and the cytoplasm. To test for the importance of this dynamic distribution, we created transgenic flies with Blanks-constructs that harbored an additional NLS in the context of a wild type or a mutant dsRBD2. The additional NLS prevented the protein from rescuing the male fertility defect of *blanks* mutant flies (Figure 5C). In the context of a mutant dsRBD2, however, the NLS-bearing transgene could rescue the fertility defect. This is consistent with a previous report that fertility is linked to the first dsRBD of Blanks while mutation of the second dsRBD has no effect (31). The first dsRBD deviates from the consensus at important RNA binding positions and thus likely engages in protein-protein, rather than protein-RNA, interaction. We measured a dissociation constant of almost 700 nM for the Blanks protein with an unchanged dsRBD1 but mutated dsRBD2, a residual affinity that may be equivalent to a null mutation as indicated by the phenotypes we observed *in vivo* (see e.g. Table 1 and Supplementary Figure S6). The reduced miRNA biogenesis observed with our NLS-Blanks transgene (with a functional dsRBD2) may result from the experimentally induced, exaggerated nuclear retention of hairpin-RNA precursors. Cytoplasmic products of some hairpin-RNAs are important for male fertility (59) and several cognate mRNAs—mainly from proteins in the metabolic pathway—are indeed misregulated in the absence of Blanks (60).

An alternative hypothesis for the molecular role of *blanks* in male fertility has recently been proposed: Blanks may foster the expression of *kl-3*, a Y-chromosome gene with very long introns that is required for male fertility. Although the particular AU-rich satellite sequence of the long introns has a potential to fold back and form stretches of structured RNA, it is currently unclear whether Blanks directly binds to this transcript or whether it participates in a protein-interaction network (61). We did not detect any relevant amounts of siRNAs that arise from the *kl-3* locus, which does not harbor a convergent transcription arrangement, in testes or S2-cells (data not shown).

Precursors for pre-miRNAs are exported by Exportin-5, which recognizes a short (∼16 nt) duplex RNA structure and a single-stranded 3′-overhang (62–64). Since the hairpins for pre-miRNAs do not require *blanks* for their export, we propose that Blanks predominantly facilitates export of long dsRNAs generated in the nucleus. This role is reminiscent of the mammalian dsRBD proteins NF90/ILF3, Staufen as well as ADAR1 and their dsRNA-dependent association with Exportin-5 or Transportin-1 (65–69). A previous screen classified *blanks/CG10630* along with other standard RNAi factors (29). We note, however, that the screen employed stable RNAi-inducing transgenes and thus a nuclear-generated dsRNA trigger. In summary, we conclude that the activity of *blanks* in spermatogenesis and dsRNA trafficking can be functionally separated via mutations in the first and second dsRBD, respectively.

Which biological role could blanks-dependent siRNAs play? Although we identified a substantial number of loci that generate blanks-dependent siRNAs, their abundance at each site is low and we do not expect a biologically relevant effect on the abundance of the corresponding mRNAs. Furthermore, the major *blanks* mutant phenotype is male infertility caused by a genetically and biochemically distinct phenomenon. The nascent transcripts that continue beyond the polyA-site are short-lived; in contrast, Ago2-loaded siRNAs are stable and the bepsiRNAs can thus help us to describe the ephemeral transcription beyond annotated 3′-ends. Yet, while such ‘fossils’ of non-coding transcription may be informative for us, they probably present no advantage to the organism.

Both *blanks* and its paralog *CG12493* are genes that recently emerged. They show less conservation than e.g. *loqs* (Supplementary Figure S7). Perhaps they arose as novel players in the arms race between viruses and flies? A proposed *Drosophila* systemic RNAi pathway relies on reverse transcription of viral RNA by retrotransposon-derived reverse transcriptases. The resulting DNA then serves as template for the generation of dsRNA, which is disseminated via exosomes to non-infected cells (70). If this pathway does indeed deliver biologically relevant amounts of siRNAs, it is conceivable that the transcription occurs in the nucleus of hemocytes where *blanks* is expressed and can facilitate the export of dsRNA. Further research is required to determine whether *blanks* mutant animals have additional phenotypes under specific circumstances, such as viral infection. There is also a striking similarity between the sperm individualization defect of blanks mutants and the RNAi-dependent sex-ratio distortion rescue by *Nmy/Tmy* siRNAs in *Drosophila simulans* (71). A comparable hpRNA system does not exist in *Drosophila melanogaster*, but it is conceivable that the processivity region with its expanded introns (61) is a ‘nascent’ hpRNA locus that may eventually develop into a *blanks*-dependent and Y-chromosome specific phenotype.

Our observations are compatible with the notion that dicing of dsRNA is more efficient in, if not limited to, the cytoplasm. However, dsRNA export from the nucleus is not entirely dependent on *blanks*, as demonstrated by the widespread application of transgenic RNAi in fly tissues that express only low levels of Blanks. Furthermore, the endo-siRNAs that target transposable elements in a constitutive manner also did not show a particular requirement for *blanks* in our analysis. Thus, alternative export routes for dsRNA must exist. For example, the end-specific association of Exportin-5 with structured RNAs (72) or simply the nuclear envelope breakdown during cytokinesis of cycling cells could suffice in certain cases. Alternatively, at least the transgenic dsRNA forming transcripts could be exported via the mRNA pathway since they carry an intron and a polyadenylation signal. This could also be possible for the transposon-targeting siRNAs if one postulates independent transcripts for sense and antisense orientation, rather than convergent transcription in *cis*. Formation of dsRNA then only occurs upon arrival in the cytoplasm, perhaps similar to our hypothesis on the biogenesis of *blanks*-independent *cis*-NAT siRNAs derived from overlapping 3′-UTRs (see above and Supplementary Figure S1).

The *CG12493* gene is likely a paralog to blanks in *Drosophila*. The gene structure and the two dsRBDs in each protein correspond very well to each other, while the unstructured N-terminus differs considerably. *CG12493* has an expression pattern that is almost identical to *blanks* except its complete absence from S2-cells (Flybase and our own unpublished data). Since the bepsiRNAs we describe are very similar between testes and S2 cells yet CG12493 is only present in the testes sample, any functional redundancy between the two proteins does not seem to include bepsiRNA biogenesis.

It is an important question whether dsRNA trafficking proteins also exist in vertebrate species, since cytoplasmic dsRNA triggers a strong interferon response and apoptosis in mammals. Protein sequence-based homology searches for orthologues of Blanks must be interpreted with caution as they are dominated by the tandem dsRBD domain structure. Yet, the structure-based homology prediction tool HHpred (73) identifies not only the Dicer co-factor TRBP, but also the NF90/ILF3 protein as a putative ortholog. NF90 harbors two dsRBDs, an RGG domain towards the C-terminus and a dimerization zinc finger domain at the N-terminus. The sequence homology between *Drosophila* Blanks and mammalian NF90 is restricted to the two dsRBDs and a basic stretch preceding the first dsRBD. Of note, the first dsRBD instance in NF90 also contributes only little to dsRNA binding (47), despite conservation of the two critical lysine residues that deviate from the consensus in the corresponding dsRBD of *Drosophila* Blanks. For NF90, this may help to discriminate longer from shorter stretches of dsRNA (47). Just like Blanks in our study, NF90 can inhibit miRNA biogenesis by trapping pre-miRNAs in the nucleus (74). NF90 modulates the host cell response to RNA virus infections (75) and viral infection also changes the biogenesis of host cell circular RNAs by reverse splicing: NF90/ILF3 was identified in a screen for circRNA biogenesis factors. Further experiments can indicate whether *Drosophila blanks* may play a role in circRNA biogenesis and whether certain functions of NF90/ILF3 can be linked to nucleo-cytoplasmic trafficking of structured RNA in mammalian cells.

## DATA AVAILABILITY

The sequencing data has been deposited at the European Nucleotide Archive (ENA) with the accession number PRJEB32123. The proteomic data is available at ProteomeXchange with the identifier PXD013792.

## Supplementary Material

gkaa072_Supplemental_Files
