# Supplementary material for: Trafficking of siRNA precursors by the dsRBD protein Blanks in Drosophila
Source: Nucleic Acids Res. 2020 Feb 6;48(7):3906–21. doi: 10.1093/nar/gkaa072 (PMC7144943; doi:10.1093/nar/gkaa072)
Supplement: gkaa072_Supplemental_Files [file gkaa072_supplemental_files.zip › Nitschko_Supplement_191220.pdf]

Supplementary Information

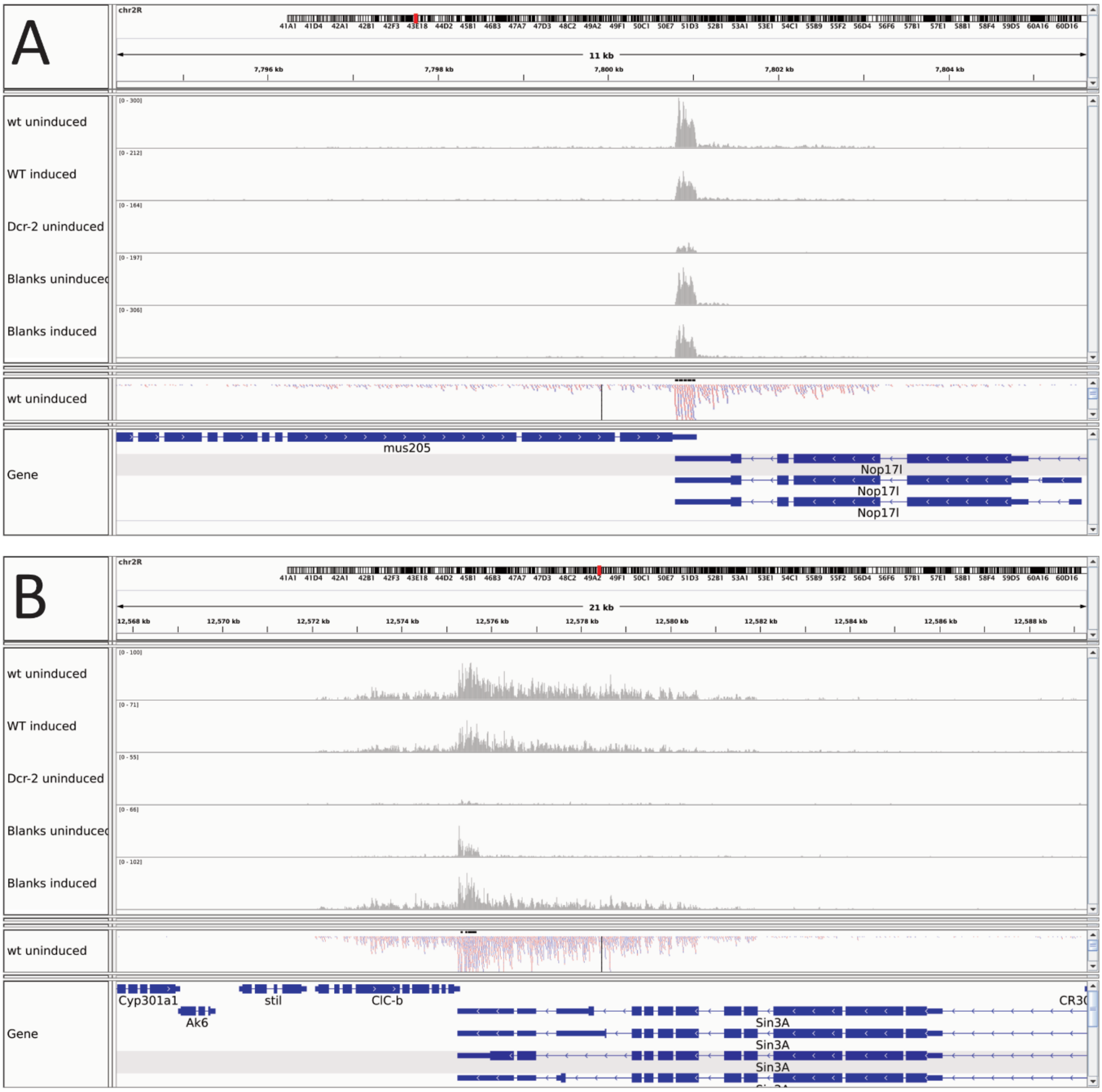

Supplementary Figure 1: cisNAT-loci can generate *dcr-2*- but not *blanks*-dependent siRNAs where the 3'-UTR regions overlap

A) The *mus-205* and *Nop17l* genes generate abundant, blanks-independent siRNAs from the overlapping 3'-UTRs. This region is nonetheless surrounded by a larger window, where *blanks*-dependent siRNAs are generated at a low abundance.

B) At the *ClC-b* and *Sin3A* locus, the relative amounts of *blanks*-independent and *blanks*-dependent siRNAs are in a comparable range, the two distinct populations are thus easier to visualize.

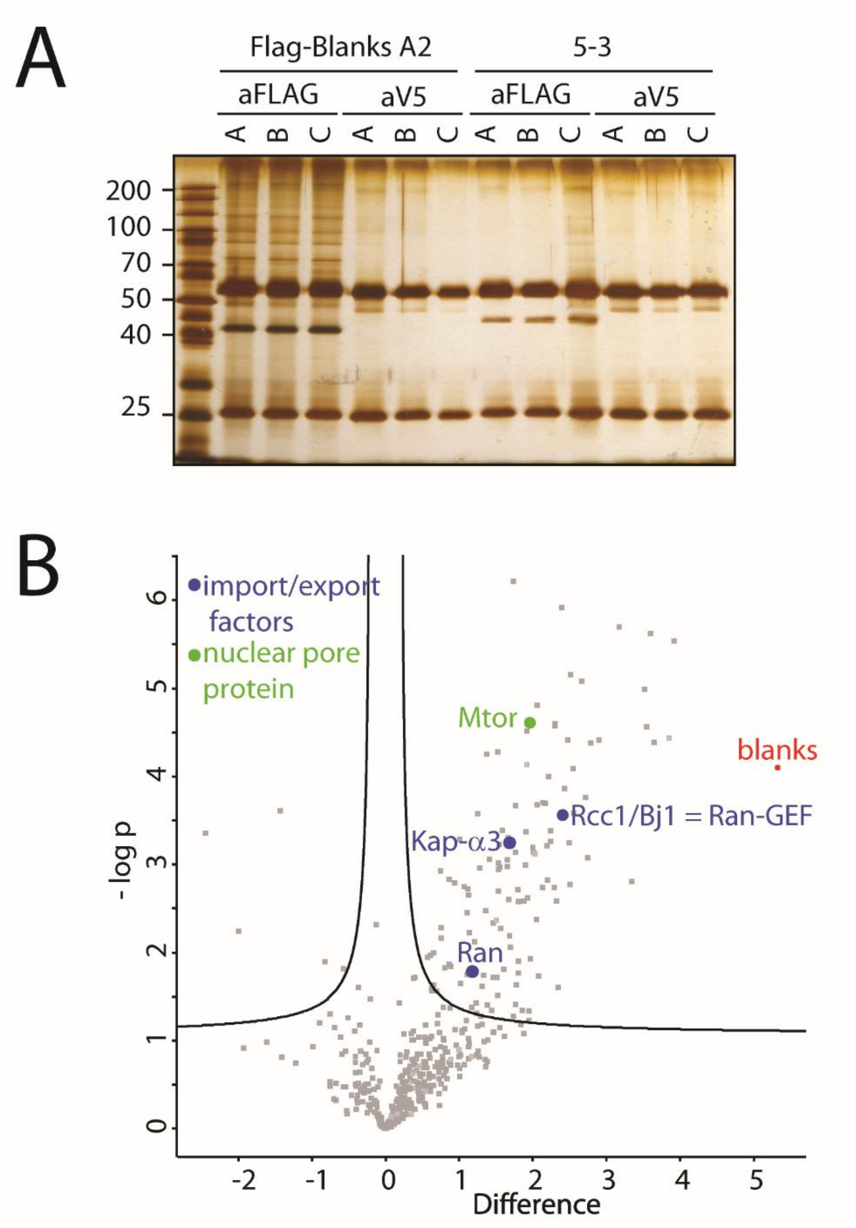

Supplementary Figure 2: Mass spectrometry-based analysis of blanks interacting proteins

A) Silver-stain of the Flag-Blanks and control immunoprecipitations.

B) Volcano plot showing the difference between the anti-Flag-Blanks and anti-V5 (control) immunoprecipitations, solid lines indicate the regions of significantly enriched proteins. Statistical analysis was performed with MaxQuant/Perseus using the adjusted t-Test with parameters FDR=0.05 and s0=0.1; significant factors involved in nucleo-cytoplasmic transport are highlighted, the complete data for all immunoprecipitations is included as Supplementary Table 3.

Detailed information on the mass spectrometry-based immunoprecipitation experiments:

S2 cells were cultured in the presence of 200 µM $CuSO_4$, harvested, washed twice with 1x PBS and resuspended in an appropriate volume of 1xPBS to obtain 10^7 cells / ml. 37 % formaldehyde stock solution was added to the suspension to reach a final concentration of 0.1% formaldehyde. The cell suspension was incubated for 5 min at room temperature on a

rotating wheel. To quench the reaction, glycine (stock 1.25 M) was added to a concentration of 125 mM, the suspension was centrifuged and the supernatant discarded.
For lysis, the cells were resuspended in an appropriate volume of lysis buffer (150 mM KAc pH7.4, 30 mM Hepes pH 7.4, 5 mM MgAc, 1 mM DTT, 15% glycerol, 1% tergitol, per 10 ml: 1 tablet Protease Inhibitor (complete EDTA-free, Roche)). Cells were mechanically lysed using the Bioruptor (Diagenode, 20 cycles a 20'' ON, 40'' OFF). Insoluble debris was pelleted by centrifugation, the supernatant represented the protein extract. For immunoprecipitation, 20 µL Dynabeads Protein G (Invitrogen) were washed with lysis buffer, resuspended in 200 µl in lysis buffer and incubated with 2 µl anti-FLAG M2 (Sigma) or anti-V5 (Biorad) antibody for 30 min at 4°C on a rotating wheel. Cell extract containing a total of 5 mg protein was added to the beads and incubated for 1 h at 4°C. The beads were washed twice with buffer 1 (150 mM KAc pH 7.4, 30 mM Hepes pH 7.4, 5 mM MgAc, 0.1 % tergitol-type NP40) and twice with buffer 2 (150 mM KAc pH 7.4, 30 mM Hepes pH 7.4, 5 mM MgAc).
The beads were either analyzed via SDS-PAGE and silver staining or via mass spectrometry. For the latter method, the beads were additionally washed 3 times with 50 mM ABC (ammonium bicarbonate) and resuspended in 100 µL of 5 ng/µl trypsin (NEB) in 1 M urea, 50 mM ABC. The digestion reaction was incubated for 30 min at 25°C, shaking at 800 rpm. The supernatant was saved, beads were washed twice with 50 µL of 50 mM ABC and finally, the supernatant was combined with the wash fractions. After the addition of DTT to a final concentration of 1 mM, the reaction was incubated at 25°C over night, shaking at 800 rpm. On the next day, 10 µL of 5 mg/ml iodoacetamide was added and the solution was incubated for 3 min in the dark at 25°C. 1 µL of 1 M DTT was added to the reaction, incubated for 10 min and in the end 2.5 µL trifluoroacetic acid was added to stop the reaction. The samples were lyophilized using a vacuum centrifuge.
LC-MS/MS was performed on an EASY-nLC 1000 chromatography system (Thermo Scientific, Waltham, MA, USA) connected to an Orbitrap XL instrument (Thermo Scientific). Peptides were diluted in 10 µl 0.1% formic acid (FA), transferred on a trap column (PepMap100 C18, 75 µm × 2 cm, 3 µm particles, Thermo Scientific) at a flow rate of 5µl/min and separated at a flow rate of 200 nL/min (Column: PepMap RSLC C18, 75 µm × 50 cm, 2 µm particles, Thermo Scientific) using a linear gradient from 2% to 35% solvent B (0.1% formic acid, 100% ACN) in 60 min. For data acquisition, a top five data dependent CID method was used. MS spectra were acquired from 300-2000 m/z at a resolution of 60.000. Collision-induced dissociation was performed using normalized collision energy of 35%. Mass spec data was analyzed using MaxQuant v1.5.3.8 and Perseus v1.5.2.4: The data that was recorded by the mass spectrometer was analyzed using MaxQuant and its label-free quantification algorithm. After removing identified known contaminants and by the algorithm speculatively identified proteins, the resulting protein levels were transferred to logarithmic values and normalized to the z-score of the sample. Proteins that were not identified or only to such a small amount that no quantification was possible were assigned imputed values. These values were randomly calculated from a normal distribution of values that are close to the normalized values of the sample. The downstream analysis was performed with data prepared in that way.

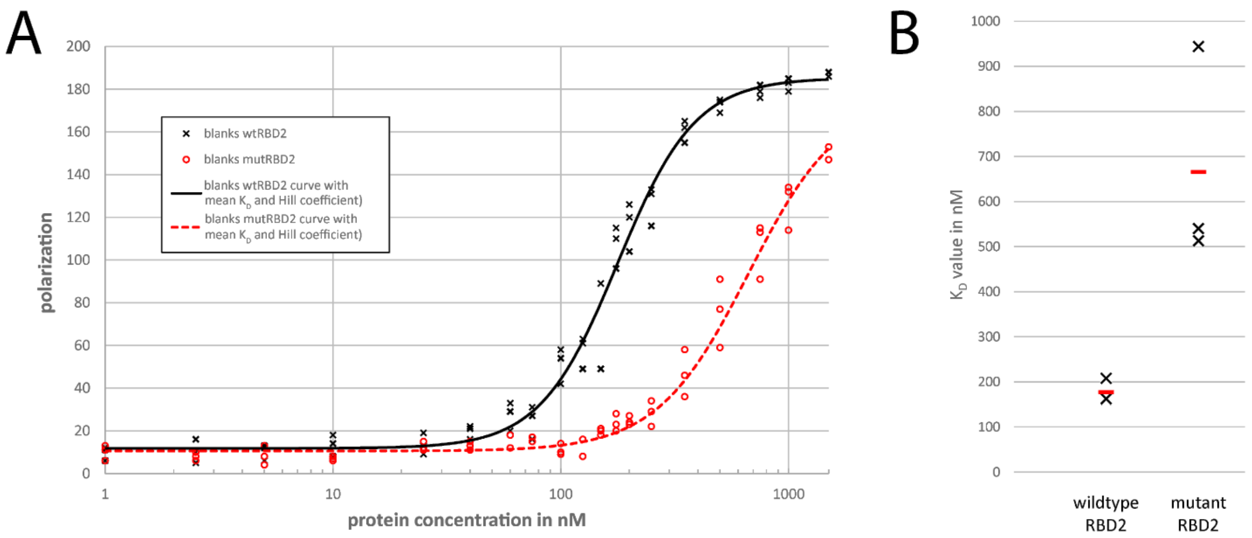

Supplementary Figure 3: RNA binding by recombinant blanks proteins *in vitro*

A) Fluorescence anisotropy measurements with wild-type (black) and dsRBD2-mutant Blanks protein (red); three independent measurements were performed.

B) We calculated a K_D-value for each of the individual measurement sets, the resulting mean and standard deviation are mentioned in the manuscripts. Here we depict the individual measurement values (x) and the resulting mean (red bar).

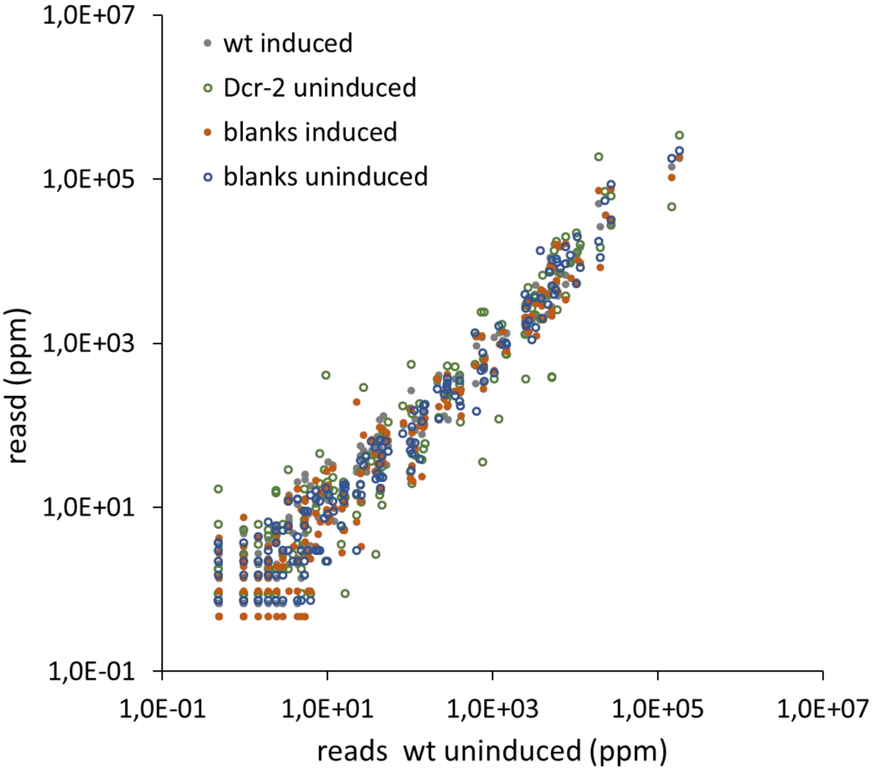

Supplementary Figure 4: miRNA abundance analysis in genome-edited S2-cells
We did not detect major changes in the miRNA profile when comparing cells with and without expression of *blanks*.

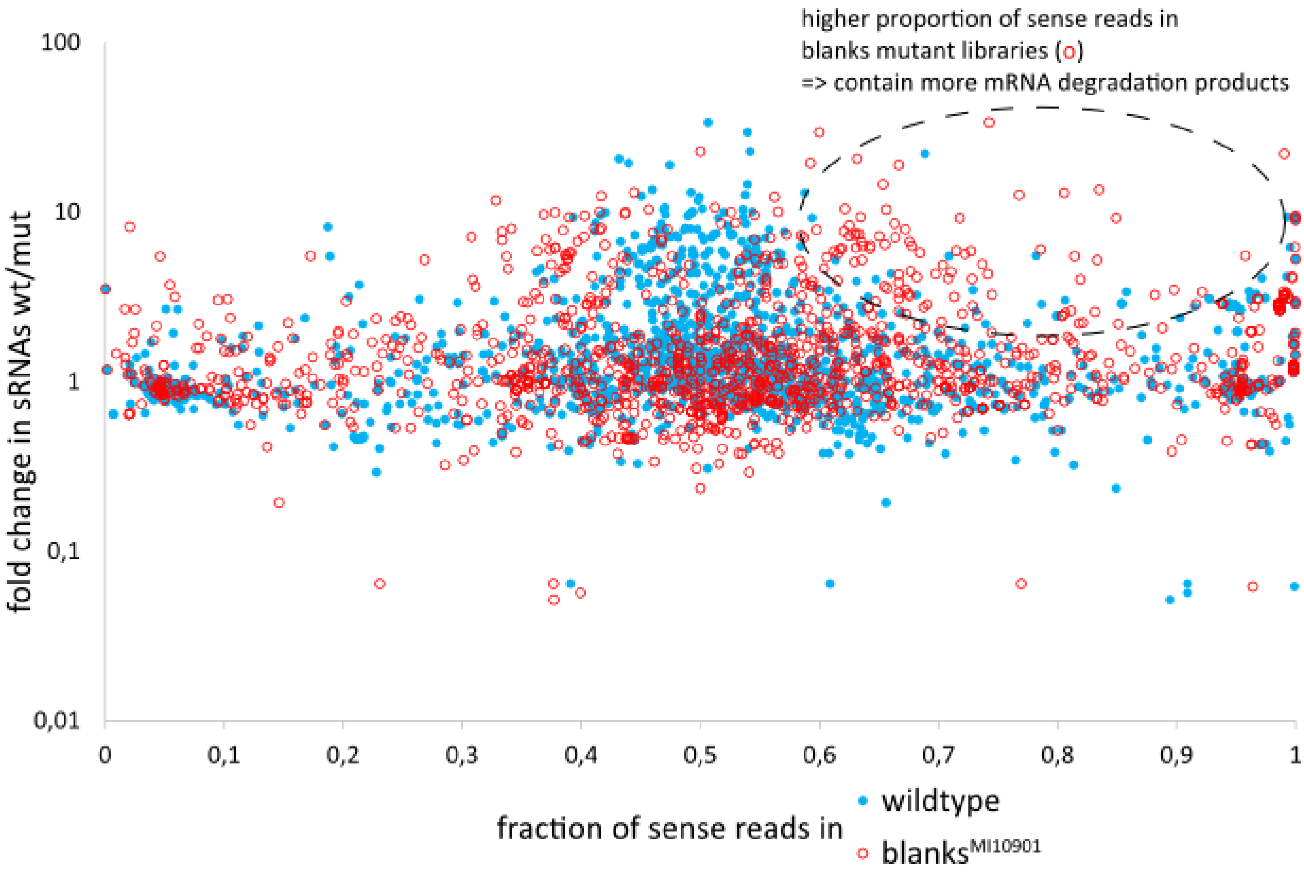

Supplementary Figure 5: bepsiRNAs derive from a dsRNA precursor
Each bepsiRNA-locus is represented as a separate datapoint. The graph shows the fold change between mutant flies and those rescued with a wild-type transgene for a given bepsiRNA locus (ordinate). In addition, the data is displayed as a function of the fraction of sense reads at the locus (abscissa). For the blue dots, the fraction sense was calculated based on the wt library – these are the same datapoints as shown in Fig. 7B of the manuscript. In this figure, we overlay the data with the same fold change values but the fraction of sense reads was calculated based on the ratios in the mutant library. In the latter, one can predict that “true” bepsiRNAs are scarce and that the remaining sequence reads have a higher content of RNA degradation products. Indeed, the datapoints redistribute away from the central region of roughly equal sense and antisense orientation, consistent with the notion that at least some of the 21-mers now represent degradation products, rather than *bona fide* siRNAs.

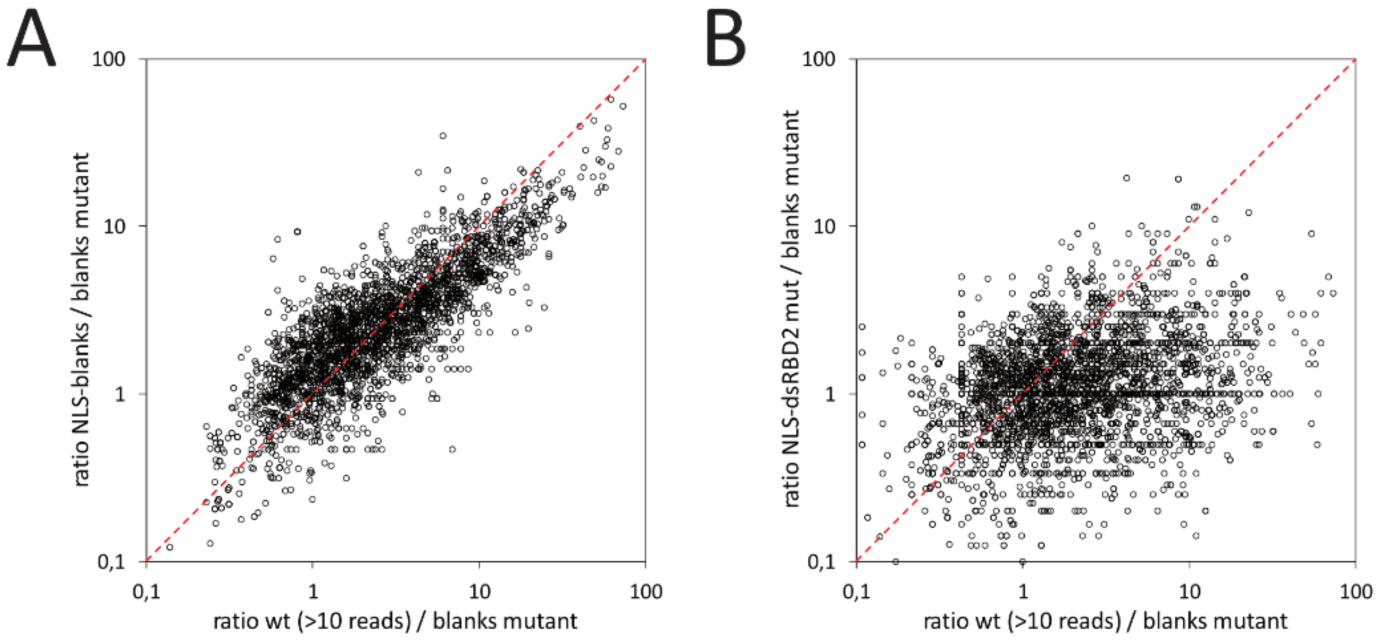

Suppl. Figure 6: A functional dsRBD2 is required for bepsiRNA production.
To clearly display the correlation, we limited the axis to 0,1 – 100 in these graphs; this led to the clipping of a small number of data points. In particular, we note that the *CG10508* gene generated a very high number of siRNAs only in the library from the wild-type blanks rescue (sense and antisense). Furthermore, the annotated hairpin-RNA CR32205 (a *pncr009* family member targeting *825-oak* family genes) produced a large number of reads only in the *blanks* mutant (sense only). This was not seen in the S2-cell data and we refrain from proposing any hypothesis why these genes stand out.

A) This plot compares the induction of bespiRNAs in the flies rescued with a wild-type transgene (abscissa) with the induction in the flies rescued with the NLS-transgene (ordinate). A clear correlation can be observed, incidating that bepsiRNAs can be made in the presence of NLS-blanks only. However, in particular the highly abundant bepsiRNAs appear to be of lower abundance.

B) The induction of bepsiRNAs in flies rescued with the NLS-dsRBD2-mutant transgene is very inefficient. Correlation is essentially lost, indicating that the dsRBD2 mutant cannot sustain the production of bepsiRNAs.

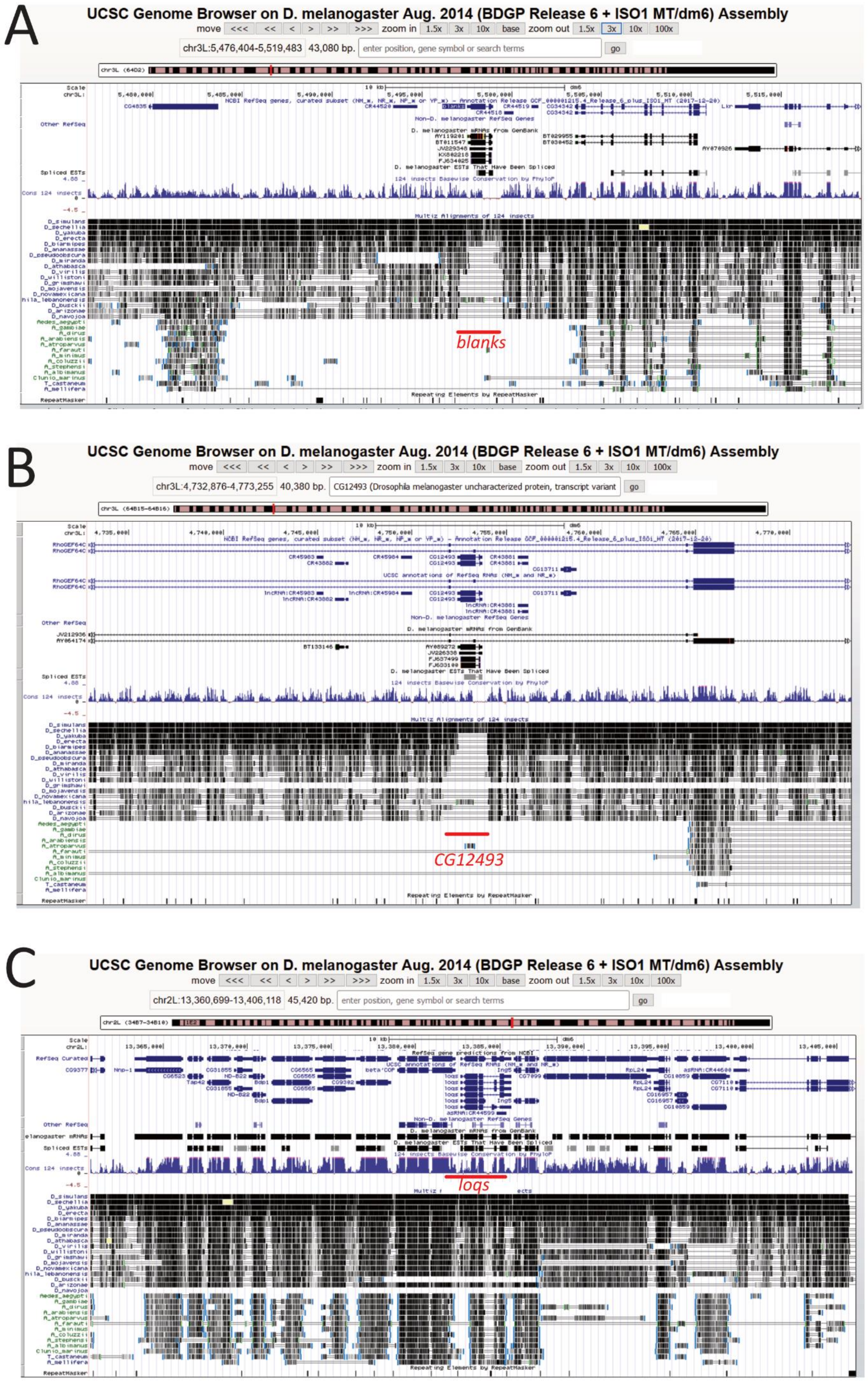
A
UCSC Genome Browser on D. melanogaster Aug. 2014 (BDGP Release 6 + ISO1 MT/dm6) Assembly
chr3L:5,476,404-5,519,483 43,080 bp.
enter position, gene symbol or search terms
blanks
B
UCSC Genome Browser on D. melanogaster Aug. 2014 (BDGP Release 6 + ISO1 MT/dm6) Assembly
chr3L:4,732,876-4,773,255 40,380 bp.
CG12493 (Drosophila melanogaster uncharacterized protein, transcript variant
CG12493
C
UCSC Genome Browser on D. melanogaster Aug. 2014 (BDGP Release 6 + ISO1 MT/dm6) Assembly
chr2L:13,360,699-13,406,118 45,420 bp.
enter position, gene symbol or search terms
loqs

Supplementary Fig. 7 (prev. page): The *blanks* and *CG12493* genes appear to have recently emerged.
We intentionally display a rather large window surrounding the genes in these UCSC genome browser views, unfortunately this results in label sizes that are too small to be readable. We ask the readers to focus on the black shading, which represents evolutionary conservation in a series of *Drosophila* genomes (blue labels one the left) and the more distantly related mosquito species (green labels). The *blanks* (A) and *CG12493* (B) genes can only be identified in closely related species, while the *loqs* gene (C), with a similar protein product, appears well conserved.

Supplementary Table 1: [accompanying Excel-File]
Primer sequences that were used for the CRISPR-based endogenous tagging and the creation of the plasmids that were used in the study.

Supplementary Table 2: [accompanying Excel-File]
List of blanks-dependent siRNA generating genomic loci compiled in the analysis of the S2 cell sequencing data. An arbitrary cutoff of the normalized read ratio of ≥5 between the wt uninduced and the *blanks* shutdown library was applied. The list consists of 638 pairs of convergent genes which represent 528 distinct loci (one transcript can converge with multiple other transcripts).

Supplementary Table 3: [accompanying Excel-File]
Detailed data tables resulting from the mass spectrometry experiment that are shown in Supplementary Figure 2.

Supplementary Table 4: [accompanying Excel-File]
List of blanks-dependent siRNA generating genomic loci compiled in the analysis of the sequencing data from testes of the *Drosophila* transgene. An arbitrary cutoff of the normalized read ratio of ≥5 between the wt rescue and the *blanks*^{MI10901} mutant library was applied. The list consists of 775 pairs of convergent genes which represent 600 distinct loci (one transcript can converge with multiple other transcripts).
